# Assessment of Anesthetic Depth Through EEG Mode Decomposition Using Singular Spectrum Analysis

**DOI:** 10.3390/s26041212

**Published:** 2026-02-12

**Authors:** Haruka Kida, Tomomi Yamada, Shoko Yamochi, Yurie Obata, Fumimasa Amaya, Teiji Sawa

**Affiliations:** 1 Department of Anesthesia, Fukuchiyama City Hospital, Kyoto 620-0056, Japan; kidah@koto.kpu-m.ac.jp; 2 Department of Anesthesiology, Graduate School of Medical Science, North Medical Center, Kyoto Prefectural University of Medicine, Kyoto 629-2261, Japan; 3 Department of Anesthesiology, Graduate School of Medical Science, Kyoto Prefectural University of Medicine, Kyoto 602-8566, Japan; t5omomi@koto.kpu-m.ac.jp (T.Y.); y-syoko@koto.kpu-m.ac.jp (S.Y.); ama@koto.kpu-m.ac.jp (F.A.); 4 Department of Anesthesiology, Yodogawa Christian Hospital, Osaka 533-0024, Japan; 3120033@ych.or.jp; 5 Hospital of Kyoto Prefectural University of Medicine, Kyoto 602-8566, Japan

**Keywords:** electroencephalogram, general anesthesia, depth of anesthesia, Hilbert–Huang transform, singular value decomposition, singular spectrum analysis

## Abstract

**Highlights:**

**What are the main findings?**

- Singular spectrum analysis (SSA) combined with Hilbert transform enabled robust, high-resolution decomposition of non-stationary EEG signals during sevoflurane general anesthesia, effectively separating trends, rhythmic components, and fast activity without predefined frequency bands.
- SSA-derived intrinsic mode function (IMF) parameters showed strong correlations with the BIS, and multiple linear regression models using selected IMF center frequencies and total power accurately predicted BIS values during the transition from maintenance to emergence (*R*^2^ ≈ 0.88).

**What are the implications of the main findings?**

- SSA offers a physiologically grounded and temporally precise alternative to conventional Fourier-based EEG analysis for depth-of-anesthesia monitoring, overcoming limitations related to non-stationarity and fixed frequency assumptions.
- This approach has the potential to improve real-time, individualized anesthesia management and provides a scalable framework into which advanced modeling or AI techniques could be integrated to enhance patient safety and reduce the risk of over- or under-anesthesia.

**Abstract:**

(1) Background: Electroencephalography (EEG) is widely used to monitor the depth of anesthesia; however, conventional Fourier-based analyses are limited in their ability to characterize non-stationary anesthetic-induced EEG dynamics. In this study, we investigated the utility of singular spectrum analysis (SSA) combined with the Hilbert transform for extracting physiologically meaningful EEG features under sevoflurane general anesthesia. (2) Methods: Frontal EEG data from ten patients undergoing sevoflurane anesthesia were analyzed from the maintenance phase through emergence. Using SSA, short EEG segments were decomposed into six intrinsic mode functions (IMFs) without pre-specified basis functions or frequency bands. Hilbert spectral analysis was applied to each IMF to obtain instantaneous frequency and amplitude characteristics. (3) Results: The SSA-based decomposition clearly captured phase-dependent EEG changes, including *α* spindle activity during maintenance and increasing high-frequency components preceding emergence. Multiple linear regression models incorporating IMF center frequencies and total power demonstrated strong correlations with the bispectral index (BIS), achieving high predictive accuracy (*R*^2^ = 0.88, MAE < 4). Compared with conventional spectral approaches, SSA provided superior temporal resolution and stable feature extraction for non-stationary EEG signals. (4) Conclusions: These findings indicate that SSA combined with Hilbert analysis is a robust framework for quantitative EEG analysis during general anesthesia and may enhance real-time, individualized assessments of anesthetic depth.

## 1. Introduction

In patients under general anesthesia (GA), electroencephalography (EEG) results change significantly due to the hypnotic effects of anesthetic drugs, causing these patients to transition from an awake state to a state of unconsciousness [1,2,3,4]. As a result, monitoring and quantifying these changes in EEG results have facilitated the development of technologies to assess the depth of anesthesia, aiding in its management [5,6,7]. Since the late 1990s, commercial devices enabling the spectral analysis of EEG results have become available, such as the bispectral index (BIS) monitor (Medtronic, Dublin, Ireland) and SEDLine (Masimo, Irvine, CA, USA). These devices use fast Fourier transform technology to detect changes in the frequency components of EEG results, calculate parameters related to the depth of anesthesia, and visualize the hypnotic effects of general anesthetics through changes in the power spectrum presented as density spectral array color maps. However, techniques that analyze changes in the frequency components of electroencephalographic signals during general anesthesia and translate them into quantitative assessments of anesthetic depth are still not fully established, as they are influenced by variables such as the type of anesthetic agent and patient age and thus continue to represent an area with considerable potential for further development.

While the Fourier transform is a powerful tool for analyzing stationary signals, it falls short for non-stationary signals. In such cases, the discrete wavelet transform is often used. This method, like the Fourier transform, decomposes data based on a basis set and is used in multi-resolution analysis [8]. However, with wavelet transforms, the time and frequency resolution changes depending on the scale of the basis function, and it is necessary to appropriately choose the type of wavelet and parameters (such as the number of scales and the range of analysis); this often requires experience and extensive trial and error, representing a significant drawback in EEG analysis.

Recently, techniques combining mode decomposition and the Hilbert transform have been investigated as new alternatives to the fast Fourier transform. EEG is considered to show the collection of micro-electrical activities of neuronal groups in the cerebral cortex. Mode decomposition methods appropriately decompose these electrical activities, capturing them as the activity of virtual functional groups of cells and observing their modes. Moreover, the Hilbert transform is a signal processing technique in the complex domain that shifts the phase by 90 degrees to obtain the instantaneous amplitude (IA) and frequency (IF). The Hilbert–Huang Transform, which combines empirical mode decomposition (EMD) with the Hilbert transform, has been reported to be effective in analyzing non-stationary time series and impulse data [9]. We previously published research in which we applied the Hilbert–Huang Transform to EEGs during propofol-induced GA [10]. Furthermore, we have evaluated the usefulness of various mode decomposition methods for EEG analysis during sevoflurane total anesthesia, including variational mode decomposition (VMD) [10,11], which decomposes EEGs into narrow-band mode functions, and the empirical wavelet transform (EWT), which uses Meyer wavelets to decompose EEGs into narrow frequency bands and assess them as precise band-pass filters for EEG decomposition [12,13].

In this study, we introduce a novel method for mode decomposition of time series data using singular spectrum analysis (SSA) [14,15]. This method is particularly suitable for non-stationary signals and does not require the pre-specification of bases. SSA involves transforming one-dimensional time series data into a matrix via a one-to-one mapping and then applying singular value decomposition (SVD) to the matrix [16]. SVD extends the spectral decomposition, which involves eigenvalues and eigenspectra of Hermitian matrices in linear algebra, to arbitrary rectangular matrices. SSA involves the use of SVD to extract trends, periodic components, and noise from data, such as time series, demonstrating its strength in analyzing non-stationary signals. In this research, we combine mode decomposition via SSA with the Hilbert transform for frequency analysis to investigate its utility for extracting EEG characteristics during GA with inhalational sevoflurane.

## 2. Theoretical Background, Methods, and Materials

### 2.1. Algorithm for SVD

#### 2.1.1. Singular Value Decomposition (SVD) and Its Application in EEG

Let $M\in K^{m\times n}$ be a rectangular matrix. The adjoint matrix of M is denoted by $M^{*}$, which is obtained by transposing M and taking the complex conjugate of its elements. For real-valued matrices, the adjoint reduces to the transpose.

If there exists a pair of unit vectors $u\in K^{m}$ and $v\in K^{n}$ satisfying

(1) $$Mv=\sigma u,\;M^{*}u=\sigma v$$

for a non-negative real number σ, then σ is called a singular value of M, and u and v are called the left and right singular vectors, respectively.

The singular value decomposition (SVD) of M is given by

(2) $$M=U\sum V^{*}$$

where U and V are unitary matrices satisfying

(3) $$U^{*}U=UU^{*}=I$$

and Σ is a diagonal matrix whose nonzero diagonal elements are the singular values of M. For real matrices, unitary matrices reduce to orthogonal matrices. An m×n matrix has at most d=min(m,n) singular values, and its left and right singular vectors form orthonormal bases of $K^{m}$ and $K^{n}$, respectively.

To apply SVD to EEG data, consider an EEG voltage time series sampled at 128 Hz for 3 s (N+1=384):

(4) $${F=\{x}_{0},x_{1},x_{2},\ldots \ldots ,x_{N-1}\}$$

For singular spectrum analysis (SSA), the window length is set to K=256 (2 s). By sliding the window, L=N−K+1=128 lagged vectors are extracted to construct the trajectory (Hankel) matrix:

(5) $$X=\left(\begin{array}{cccc} x_{0} & x_{1} & \ldots & x_{K-1}\\ x_{1} & x_{2} & \vdots & x_{K}\\ \vdots & \vdots & \ddots & \vdots \\ x_{L-1} & x_{L} & \ldots & x_{N-1} \end{array}\right)$$

Each row contains K consecutive data points, and identical values appear along anti-diagonals, which is characteristic of a Hankel matrix. The columns are called L-lagged vectors and the rows are K-lagged vectors.

The trajectory matrix is decomposed using SVD:

(6) $$X=U{\sum V}^{T}$$

where U and V are unitary matrices containing left and right singular vectors of X, respectively, and Σ is a diagonal matrix whose elements are singular values $\sigma_{i}$ (i=0,…,d−1, d=min(K,L)) arranged in descending order.

Since Σ is diagonal, the decomposition can be written as a sum of rank-one matrices:

(7) $$X=\sum_{i=0}^{d-1}\sigma_{i}\;u_{i}\;v_{i}^{T}\;\equiv \sum_{i=0}^{d-1}X_{i}$$

where $u_{i}$ and $v_{i}$ are the i-th columns of U and V. Each triplet $\left\{u_{i},\sigma_{i},v_{i}\right\}$ is referred to as an eigentriple. Larger singular values indicate more dominant signal components.

For dimensionality reduction or denoising, only components with significant singular values are retained for reconstruction. In EEG analysis, these components can be grouped into physiologically meaningful categories (e.g., trend, oscillatory activity, noise) and used for feature extraction.

#### 2.1.2. Time Series Component Separation and Grouping

Up to this point, the EEG time series F has been embedded into a sequence of L-lagged vectors, thereby forming the trajectory matrix of F. SVD is then applied to this matrix, resulting in a set of elementary matrices whose sum reconstructs the original trajectory matrix. In an ideal scenario, the time series F can be decomposed into a sum of mutually separable components, $F=\sum_{j}F^{\left(j\right)}$. By appropriately grouping the corresponding elementary matrices $X_{i}$, the trajectory matrix can be expressed as

(8) $$X=\sum_{k\in S}X_{k}\;+\;\sum_{l\in T}X_{l}\;+\;\ldots =\sum_{j}X^{\left(j\right)}$$

where S and T are mutually exclusive index sets and $X^{\left(j\right)}$ represents the trajectory matrix corresponding to the component $F^{\left(j\right)}$. Each $X^{\left(j\right)}$ preserves the Hankel structure of the original trajectory matrix. The reconstructed time series $F^{\left(\tilde{j}\right)}$ is then obtained via diagonal averaging, in which the elements of $X^{\left(j\right)}$ are averaged along anti-diagonals extending from the upper right to the lower left of the matrix.

The element ${\tilde{x}}_{\left(p,q\right)}$ of the reconstructed matrix ${\tilde{X}}^{\left(j\right)}$, with s=p+q, K=256≥L=128, and s=0, 1, …,N−1, is defined by the diagonal averaging operation as follows:

(9) $${F_{S}}^{\left(j\right)}\equiv \;{\tilde{x}}_{\left(p,q\right)}=\left\{\begin{array}{c} \frac{1}{s+1}\sum_{p=0}^{s}{{\tilde{x}}^{\left(j\right)}}_{\left(p,\;\;\;s-p\right)}\;\;\;\;\;\;\;\;\;\;\;\;\;\;\;\;\;\;\;\;\;(0\leq s\leq L-2)\\ \frac{1}{L}\sum_{p=0}^{L-1}{{\tilde{x}}^{\left(j\right)}}_{\left(p,\;\;\;s-p\right)}\;\;\;\;\;\;\;\;\;\;\;\;\;\;\;\;\;\;\;\;\;\;\;\;\;\;\;\;\;\;\left(L-2\leq s\leq K-1\right)\\ \frac{1}{L+K-s-1}\sum_{p=s-K+1}^{L-1}{{\tilde{x}}^{\left(j\right)}}_{\left(p,\;\;\;s-p\right)}\;(K\leq s\leq K\;+\;L\;\;-\;2) \end{array}\right.$$

Each grouped time series, obtained by summing selected elementary components, is treated as an intrinsic mode function (IMF), to which the Hilbert transform is applied.

#### 2.1.3. Hilbert Transform

The Hilbert transform is applied to the IMF components, denoted by $imf\left(t\right)$, and the analytic signal $z\left(t\right)$ is obtained as follows [9,10,17,18,19,20,21]:

(10) $$\begin{array}{l} Analytic\;signal\;z\left(t\right)=imf\left(t\right)+iH[imf\left(t\right)]=a\left(t\right)e^{i\int \omega \left(t\right)dt}\;\;\;\;\;\;\;\;\;\;\;\;\;\;\;\;\;\;\;\;\;\;\;\;\;\; \end{array}$$

(11) $$\begin{array}{ll} \mathrm{in}\;\mathrm{which}\;a\left(t\right)=\sqrt{{imf\left(t\right)}^{2}+{H\left[imf\left(t\right)\right]}^{2}} & \;\text{Instantaneous amplitude (IA)} \end{array}$$

(12) $$\begin{array}{ll} \omega (t)={\frac{t}{dt}[tan}^{-1}\left(\frac{H[imf(t)}{imf\left(t\right)}\right)]\;\;\;\;\;\;\;\;\;\;\;\;\;\;\;\; & \text{Instantaneous frequency (IF)}\; \end{array}$$

(13) $$\begin{array}{ll} \;\;\;h\left(\omega \right)=\int H(\omega ,\;t)dt\;\;\;\;\;\;\;\;\;\;\;\;\;\;\;\;\;\;\;\;\;\;\;\;\;\;\;\;\;\;\;\;\;\;\;\;\;\; & \text{Marginal Hilbert spectrum}\;\;\;\;\;\;\;\;\; \end{array}$$

where ω(t) and a(t) are the IF and the IA of the IMF used to obtain a time–frequency distribution for signal $imf\left(t\right)$ and the Hilbert amplitude spectrum H(ω,t).

### 2.2. Anesthesia Management and Data Acquisition

This study used a previously reported [10] and publicly available dataset of EEG data from patients under sevoflurane GA (ten minutes until awakening in ten cases of GA, https://github.com/teijisw/EEG_DataSet/tree/master/general_anesth_sevoflurane, accessed on 13 December 2025) (Supplementary File S3). The original data were acquired using EEG Analyzer software [22] (EEG Analyzer ver. 54_GP, http://anesth-kpum.org/blog_ts/?p=3169, accessed on 13 December 2025) linked to a VISTA A-3000 BIS monitor with a BIS Quatro sensor [22].

The EEG signals were acquired using a BIS monitor featuring a noise-shaping sigma-delta analog-to-digital conversion system. The device operates at an internal sampling frequency of 16,384 Hz to perform oversampling for noise reduction, yielding an effective sampling frequency of 256 Hz (16-bit resolution) for internal processing and storage. It should be noted that while the standard effective rate is 256 Hz, the data may be downsampled to 128 Hz depending on the external output software settings. The recording bandwidth ranges from 0.16 Hz to 100 Hz, with integrated filtering for commercial power line noise (50/60 Hz). Designed to accommodate high skin impedance, the system features an input impedance of 50 MΩ (DC) and 5 MΩ (at 10 Hz), maintaining a low noise level of less than 0.3 µV RMS to capture weak EEG signals. The analysis is typically conducted using a 2-s epoch length.

The standard adult sensor used with the Bispectral Index Monitor is the BIS Quatro Sensor, which features a four-electrode integrated system on a single adhesive sheet. The electrodes are positioned to capture signals from specific anatomical landmarks: Electrode 1 is placed at the center of the forehead (approximately 5 cm above the nasion), Electrode 2 adjacent to it, Electrode 4 directly above the eyebrow, and Electrode 3 at the temple (between the lateral canthus and the hairline). The sensor derives a single channel of frontal EEG data by measuring potential differences from the frontal (near Fp1/Fp2) to the temporal regions (near F7/F8). Within this configuration, specific electrodes serve as ground and reference points, facilitating the identification and filtration of artifacts such as eye movements (EOG) and electromyography (EMG) noise. For more details on patient information, anesthesia management, and data acquisition, such as the raw EEG, BIS, 95% spectral edge frequency (SEF_95_), total power (TP), and absolute power derived from electromyography in the 70–110 Hz range (EMG_low_), please refer to our previous report [10].

**Figure 1:**
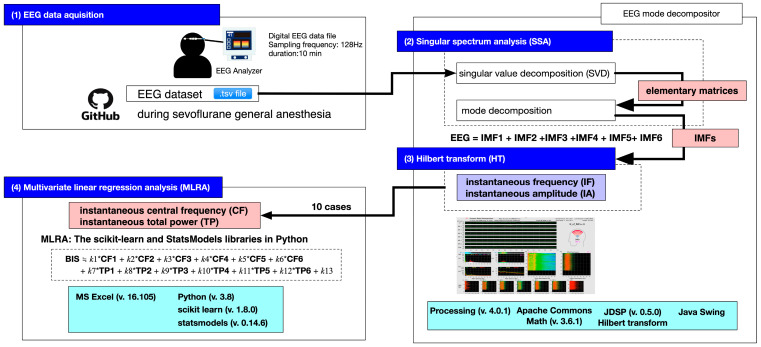
The analytical process from electroencephalogram (EEG) data acquisition during sevoflurane general anesthesia through signal decomposition to multivariate linear regression analysis (MLRA). The process consists of four stages: “(1) EEG data acquisition”, “(2) singular spectrum analysis”, and “(3) Hilbert transform”, as well as “(4) multivariate linear regression analysis”. For data acquisition, a digital EEG dataset publicly available on GitHub (https://github.co.jp, accessed on 13 December 2025) is used (sampling frequency: 128 Hz, recording duration: 10 min, format: .tsv). For signal decomposition and feature extraction, processing is performed using a GUI tool called “EEG mode decompositor,” developed in the Processing 4 (Java) environment with the Apache Commons Math and JDSP libraries. Singular spectrum analysis (SSA) employing singular value decomposition (SVD) decomposes the original EEG signal into elementary matrices and expresses it as the sum of six intrinsic mode functions (IMFs). The Hilbert transform (HT) is applied to each extracted IMF to calculate the instantaneous frequency (IF) and instantaneous amplitude (IA). In the final stage, multivariate linear regression analysis (MLRA) is performed on 10 cases in a Python environment (scikit-learn, StatsModels). A regression model predicts BIS values—an indicator of anesthetic depth—using the instantaneous central frequency (CF) and instantaneous total power (TP) of each IMF derived from the Hilbert transform as explanatory variables. The main software environments are Processing 4.0.1, Apache Commons Math 3.6.1, and JDSP 0.5.0 on the Java side, Microsoft Excel 16.105, and Python 3.8, scikit-learn 1.8.0, and StatsModels 0.14.6 on the Python side.

Figure 1 shows the analysis flowchart from EEG acquisition in general anesthesia patients, through mode decomposition and Hilbert transform, to multiple linear regression analysis. Using EEG data from 10 cases, we applied the Hilbert transform to the six IMFs obtained through mode decomposition via SSA and calculated variations over 10 min for a total of 12 parameters: instantaneous frequency (IF, represented as central frequency, CF, using the median value of 10 cases) and instantaneous amplitude (IA, represented as total power, TP). In the previously reported mode decomposition using VMD and EWT [13,20], the fast Fourier transform algorithm requires data points that are powers of two. Therefore, mode decomposition was performed on 8-s EEG epochs (128 Hz, 1024 data points, with a 4-s overlap). In contrast, the current analysis used 3-s EEG epochs (128 Hz, 384 data points, with a 1-s overlap). For each epoch, a trajectory matrix of 128 rows × 256 columns was created, and decomposition into six IMFs was performed through SSA processing. As the data volume increases, the computational load on the CPU increases due to large-scale matrix calculations. Therefore, to ensure real-time performance in terms of both computational processing and the graphical display of results, we adopted the method of repeatedly analyzing shorter EEG segments at shorter time intervals.

Finally, we performed multivariate linear regression analysis using these 12 parameters as explanatory variables and BIS values as the dependent variable.

### 2.3. Data Processing and Statistics

The changes in the various EEG parameters between two time points were compared statistically by conducting Kruskal–Wallis tests with SPSS Statistics (28.0.1.0, IBM Corp., Armonk, NY, USA), and *p*-values < 0.05 were considered statistically significant. Multiple linear regression (MLR) analysis of the BIS and IMF parameters was performed using the scikit-learn [16] and StatsModels libraries in Python (ver. 3.8), as in our recent reports [23,24].

## 3. Results

Using an EEG dataset from 10 cases of general anesthesia with sevoflurane, mode decomposition was performed using SSA. This dataset consisted of single-channel frontal EEG recordings from a BIS monitor, spanning 10 min from the maintenance phase of general anesthesia to the emergence phase.

### 3.1. Case Analysis of SSA Application

**Figure 2:**
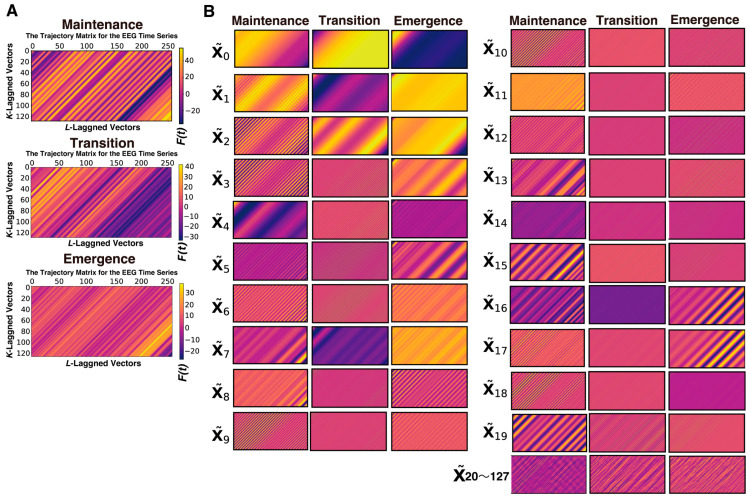
An inspection of 128 trajectory matrices of EEG time series across three phases of sevoflurane general anesthesia: (**A**) color maps of the Hankelized trajectory matrices of EEG time series before SSA decomposition and (**B**) an inspection of the first 20 and the remaining 108 elementary matrices after SSA decomposition across the three phases of sevoflurane general anesthesia.

First, we present the SSA that was conducted using the EEG data acquired from Patient #1 (a 54-year-old female who was diagnosed with IgA nephropathy and underwent palatine tonsillectomy) [10], anesthetized with sevoflurane. We analyzed 3 s long epochs of EEG data that were recorded at 128 Hz during three distinct phases of GA up to the point of emergence: the maintenance (10 min before emergence), transition (1 min before emergence), and emergence phases. From each 3-s segment containing 384 data points, a trajectory matrix of 128 rows × 256 columns was constructed using a 2-s window (256 data points), shifted by 1 data point at a time. Then, a process was applied to group the elementary matrices into several component sets and subsequently reconstruct each set into a time series (Figure 2).

A process was applied to convert each elementary matrix into a Hankel matrix and subsequently reconstruct it into a time series. The color visualization of the trajectory matrix of the EEG time series highlights the characteristics of the elementary matrices. For example, ${\tilde{X}}_{0}$ and ${\tilde{X}}_{1}$ changed very gradually throughout the entire time series and can be interpreted as trend components. ${\tilde{X}}_{2}$ and ${\tilde{X}}_{3}$ also often exhibited slow periodicity. In the range from approximately ${\tilde{X}}_{4}$ to ${\tilde{X}}_{9}$, periodicity was frequently visible, whereas from ${\tilde{X}}_{10}$ onward, the components consisted of fine waves with high frequencies.

**Figure 3:**
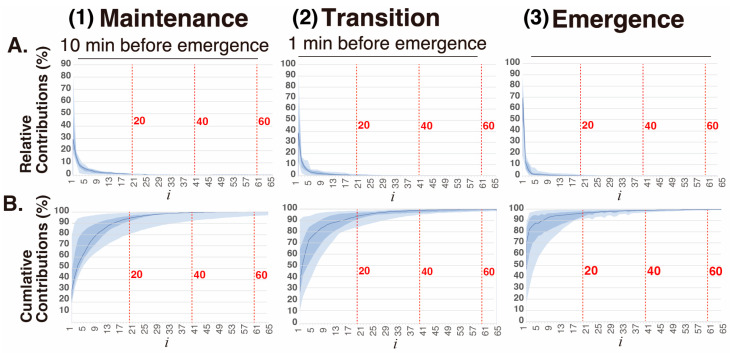
Plots of the (**A**) relative and (**B**) cumulative contributions of the 64 elementary matrices to the trajectory matrix of the EEG time series in three phases (1: maintenance, 10 min before emergence; 2: transition, 1 min before emergence; 3: emergence) of sevoflurane general anesthesia.

The relative contributions

(14) $$\frac{\sigma_{i}^{2}}{\sum_{k=0}^{d-1}\sigma_{k}^{2}}$$

and cumulative contributions

(15) $$\frac{\sum_{j=0}^{i}\sigma_{j}^{2}}{\sum_{k=0}^{d-1}\sigma_{k}^{2}}$$

of the first 64 elementary matrices $X_{i}\;$ in the decomposition X =$\;\sum_{i=0}^{d-1}X_{i}$ obtained via SVD of 3-s EEG segments in the three phases of general anesthesia (Figure 3). The elementary matrices $X_{0}$ and $X_{1}$ contributed 22% and 21%, respectively, to the overall decomposition of X. The first 20 elementary matrices together accounted for 97% of the total contribution. In this study, the 128 matrices obtained via SSA were grouped into six sets: 0–1, 2–3, 4–6, 7–9, 10–19, and 20–127. The matrices within each group were summed:

(16) $${\tilde{X}}_{0}\sim {\tilde{X}}_{1},\;{\tilde{X}}_{2}\sim {\tilde{X}}_{3},{\;\tilde{X}}_{4}\sim {\tilde{X}}_{6},\;{\tilde{X}}_{7}\sim {\tilde{X}}_{9},\;{\tilde{X}}_{10}\sim {\tilde{X}}_{19},\;{\tilde{X}}_{20}\sim {\tilde{X}}_{127}$$

(17) $${\tilde{X}}^{\left(IMF-1\right)}={\tilde{X}}_{0}+{\tilde{X}}_{1}\Rightarrow {\tilde{F}}^{\left(IMF-1\right)}={\tilde{F}}_{0}+{\tilde{F}}_{1}\;\;\;\;\;\;\;\;\;\;\;\;\;\;\;\;\;\;\;\;\;\;\;\;\;$$

(18) $${\tilde{X}}^{\left(IMF-2\right)}={\tilde{X}}_{2}+{\tilde{X}}_{3}\Rightarrow {\tilde{F}}^{\left(IMF-2\right)}={\tilde{F}}_{2}+{\tilde{F}}_{3}\;\;\;\;\;\;\;\;\;\;\;\;\;\;\;\;\;\;\;\;\;\;\;\;\;$$

(19) $${\tilde{X}}^{\left(IMF-3\right)}={\tilde{X}}_{4}+{\tilde{X}}_{5}+{\tilde{X}}_{6}\Rightarrow {\tilde{F}}^{\left(IMF-3\right)}={\tilde{F}}_{4}+{\tilde{F}}_{5}+{\tilde{F}}_{6}\;\;\;\;\;$$

(20) $${\tilde{X}}^{\left(IMF-4\right)}={\tilde{X}}_{7}+{\tilde{X}}_{8}+{\tilde{X}}_{9}\Rightarrow {\tilde{F}}^{\left(IMF-4\right)}={\tilde{F}}_{7}+{\tilde{F}}_{8}+{\tilde{F}}_{9}\;\;\;\;\;$$

(21) $${\tilde{X}}^{\left(IMF-5\right)}={\tilde{X}}_{10}+\cdots +{\tilde{X}}_{19}\Rightarrow {\tilde{F}}^{\left(IMF-5\right)}={\tilde{F}}_{10}+\cdots +{\tilde{F}}_{19}\;\;\;$$

(22) $${\tilde{X}}^{\left(IMF-6\right)}={\tilde{X}}_{20}+\cdots +{\tilde{X}}_{127}\Rightarrow {\tilde{F}}^{\left(IMF-6\right)}={\tilde{F}}_{20}+\cdots +{\tilde{F}}_{127}\;$$

where ${\tilde{F}}^{\left(IMF-i\right)}$ is an IMF time series.

Using SSA, each 3-s EEG signal was decomposed into six intrinsic mode functions (IMFs) according to the above classification. According to the rules of mode decomposition, the original EEG waveform can be reconstructed by aligning the time axes and summing the voltage values of the six IMFs. The Hilbert transform was then applied to each IMF to obtain the Hilbert spectrum.

**Figure 4:**
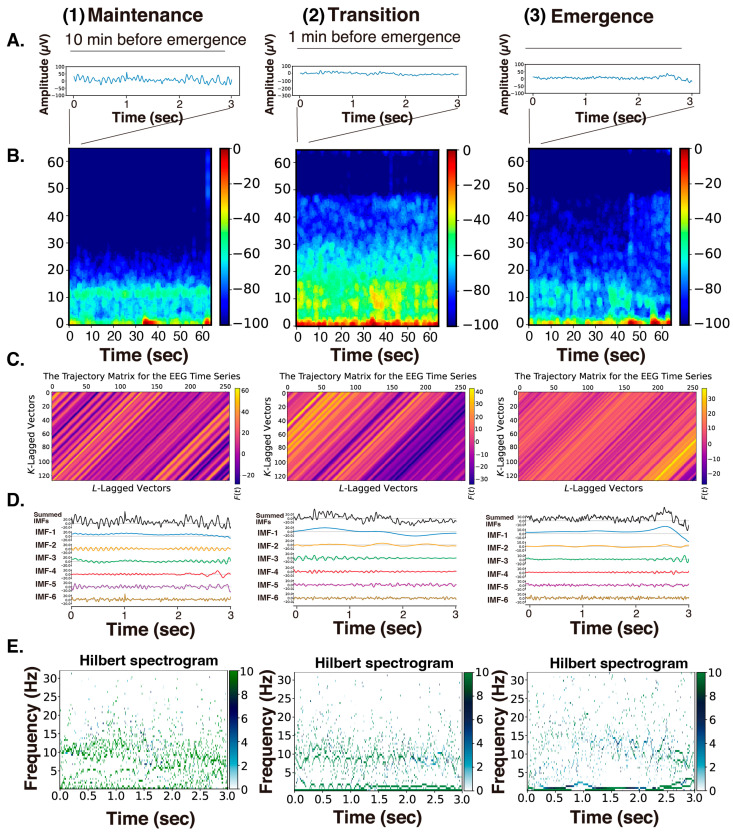
(**A**) The raw EEG data (3 s), (**B**) density spectral arrays (DSAs) (64 s, starting with a 3 s long segment of raw EEG data), (**C**) trajectory matrix images of EEG time series, (**D**) IMFs obtained over 3 s via SSA methods, and (**E**) Hilbert spectrograms across three GA phases (1: maintenance, 10 min before emergence; 2: transition, 1 min before emergence; 3: emergence) in Patient #1 (54-year-old woman, Xiaoli Li et al., 2008 [20]).

Figure 4 presents the raw EEG data (3 s), density spectral arrays (DSAs) (64 s, starting with a 3 s long segment of raw EEG data), trajectory matrix images of EEG time series, IMFs obtained over 3 s via the SSA method, and Hilbert spectrograms across these GA phases for Patient #1 (54-year-old woman, Tai Nguyen-Ky et al., 2012 [10]). During the maintenance phase of anesthesia, IMFs 1–4 were predominantly in the frequency range of around 15 Hz or lower. In the DSA during the maintenance phase, an enhancement of *α* waves characteristic of sevoflurane general anesthesia was observed, with increased power around 12 Hz. Correspondingly, spindle waves, indicative of *α* wave enhancement, were visible in the EEG waveform. In the DSA during the transitional phase, while the *α* enhancement persisted, there was an additional increase in higher-frequency components between 15 and 47 Hz. Compared to that for the maintenance phase, the EEG waveform showed reduced amplitude and the emergence of fast-frequency components.

In the awakening phase, the *α* wave enhancement disappeared, and although the fast-frequency components persisted, an overall reduction in power was observed. The EEG waveform during this phase was predominantly composed of low-amplitude *β* wave activity. When the trajectory matrices were compared across the three phases, the maintenance and transitional phases exhibited coarser diagonal striations indicative of periodicity, whereas the awakening phase showed finer diagonal patterns. The middle panels of Figure 4 display the six IMFs of the EEG signals, obtained through SSA. In the maintenance phase, IMF-2 and IMF-3 captured the spindle waves associated with enhanced *α* activity. In the transitional phase, the spindle wave in IMF-2 disappeared. In the awakening phase, IMF-1 through IMF-3 presented nearly flat waveforms, while IMF-5 and IMF-6 predominantly exhibited fast wave activity. The bottom panels of Figure 4 show the Hilbert spectrograms for each IMF over a 3-s interval, depicting the time series relationship between IA and IF determined via the Hilbert transform. In the maintenance phase, a characteristic band around 10 Hz was evident. This feature was also present around 8–10 Hz during the transitional phase. However, in the awakening phase, these characteristic bands disappeared.

### 3.2. Hilbert Spectrograms of the IMFs in the SSA Method

**Figure 5:**
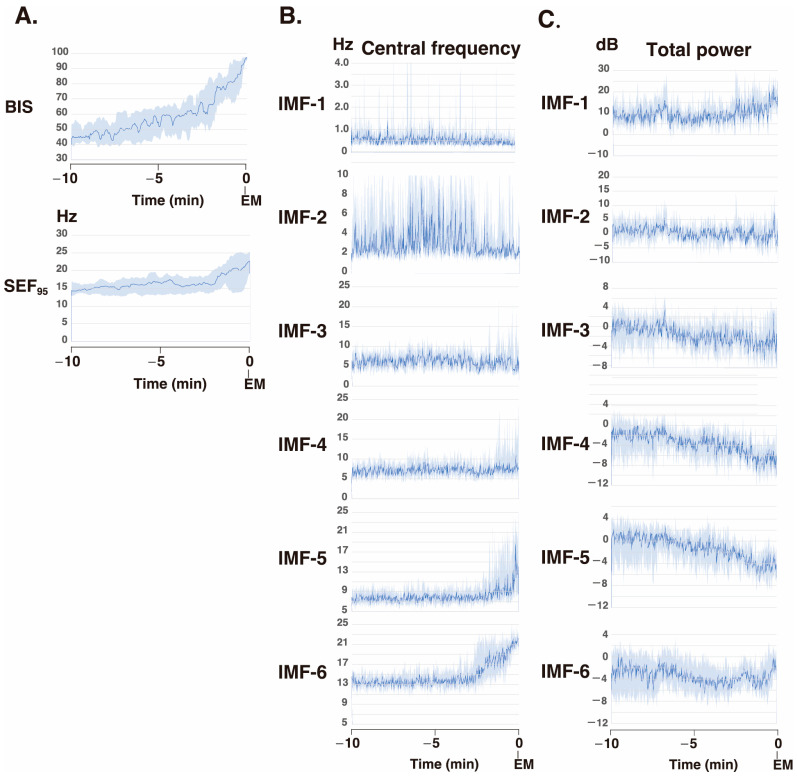
Time course changes in the (**A**) processed EEG parameters (BIS index, SEF95), (**B**) central frequencies, and (**C**) total powers of the intrinsic mode functions (IMFs) of the singular spectrum analysis (SSA) for 10 min before emergence from general anesthesia in ten patients [20]. The total power is displayed on a log scale (in decibels (dB) obtained from the power (*P*, µV^2^) using the conversion dB = 10 × log_10_*P*). The data are presented as medians (dark blue lines), and 25th–75th percentile ranges (light blue areas). BIS index: bispectral index, SEF_95_: spectral edge frequency 95%, EM: emergence.

To gain further understanding of the frequency characteristics of each IMF produced via SSA across the 10-min phase before emergence, a Hilbert spectral analysis of the IMFs was performed. This analysis assessed variations in the IF and IA values derived from the Hilbert transforms of the IMFs across each phase for all 10 patients (Figure 5). Video images (Supplementary Files S4–S13) present the analysis screens (the Hilbert spectrograms for each IMF obtained via the SSA method, the summed IMF (IMF-all, which corresponds to the original EEG data), and the DSAs for 30 min until emergence) for the 30-min period up to the awakening phase in these 10 cases.

Table 1 summarizes how SSA-derived IMF parameters changed across three periods of general anesthesia (maintenance, transition, and emergence). Overall, the CFs show a phase-dependent redistribution of spectral content, while TP tends to decrease during transition and diverge across IMFs during emergence. During the transition phase, CF1 and CF2 decrease, indicating a shift of the slowest components toward lower-frequency activity. In contrast, CF3 and CF4 increase modestly, suggesting relative stabilization or slight acceleration in the mid-frequency range. CF5 and CF6 show only small increases in transition, implying limited change in higher-frequency IMFs at this stage. In the emergence phase, the pattern becomes more pronounced. CF1 and CF2 decline further, showing a sustained reduction in the lowest-frequency IMFs. Meanwhile, CF5 and CF6 increase markedly, with CF5 shifting into the α range and CF6 rising within the β band. This indicates that, approaching awakening, the higher-frequency IMFs become more dominant in terms of their center frequency, consistent with the re-emergence of faster EEG activity. For TP, TP1 decreases in transition but increases in emergence, suggesting that the slowest IMF regains power as awakening approaches. In contrast, TP2–TP5 drop substantially during transition and decrease further during emergence, indicating progressive attenuation of these components over time. TP6 shows only moderate change, remaining relatively stable compared with the pronounced reductions observed in TP2–TP5. Taken together, the table indicates that the transition to emergence is characterized by a separation between low-frequency and high-frequency IMF behaviors: the lowest-frequency IMFs shift downward in frequency (CF1–CF2), while the higher-frequency IMFs shift upward (CF5–CF6). Simultaneously, power changes are IMF-specific, with broad reductions in several intermediate IMFs (TP2–TP5) and comparatively preserved or rebounding power in TP1 (and relatively stable TP6).

### 3.3. Multiple Linear Regression Models to Predict the BIS

Next, we analyzed the processed EEG data, sampled every 8 s, acquired 10 min before emergence from 10 patients who had been administered sevoflurane GA. We explored the relationship between the BIS (a clinical gauge of the depth of anesthesia) and the IMF parameters, including the center frequency (i.e., the average of the Hilbert spectrum) and TP, which were converted from power in *P*(µV^2^) to decibels (dB) using dB = 10·log_10_*P*. Multiple linear regression (MLR) models were developed using IMF parameters to predict the BIS using the Data_EME10s dataset. The comprehensive MLR formula included individual terms for the center frequencies and the TPs of the six IMFs, yielding a model with 12 explanatory variables.

**Figure 6:**
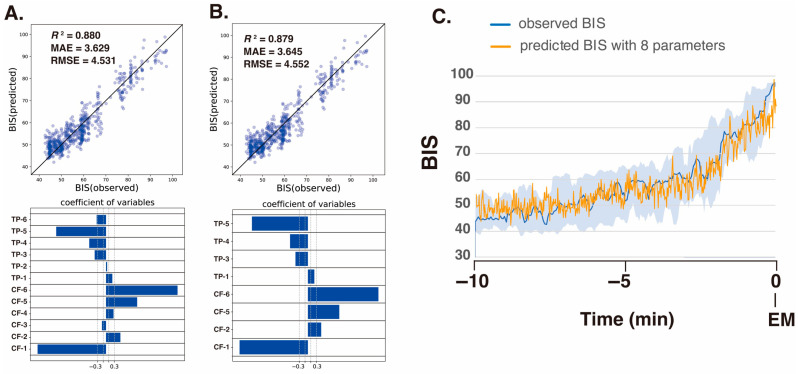
Multiple linear regression (MLR) analysis was performed with the BIS values from the BIS monitor as the objective variable and the statistically significant center frequency (CF) and total power (TP) medians of IMF-1 to IMF-6 obtained via SSA decomposition as explanatory variables for the 10 min before emergence in the ten patients [2]. (**A**) MLR with all 12 parameters of IMFs. (**B**) MLR with 8 significant parameters of IMFs. The correlation between the observed BIS and predicted BIS was calculated from the MLR and the coefficient variables. In the scatter plots, each blue data point is rendered with an alpha (opacity) of 0.3, so areas where points overlap appear in a darker shade. (**C**) The time-course changes in the observed and predicted BIS derived from MLR using 8 parameters were computed from the MLR. The data are presented as medians (dark blue lines), 25th–75th percentile ranges (light blue areas), and predicted BIS values (orange line). BIS, bispectral index; EM, emergence; TP, total power; CF, central frequency of instantaneous frequency. *R*^2^, coefficient of determination; MAE, mean absolute error; RMSE, root-mean-squared error.

The analysis yielded robust correlations, with the SSA-based MLR model using all 12 parameters (6 center frequencies and 6 TPs) demonstrating the strongest correlation (*R*^2^ = 0.880) and the lowest errors (mean absolute error: MAE = 3.629; root-mean-squared error: RMSE = 4.531) among the four models examined; the results are detailed in Table 2. Notably, in the SSA approach, the center frequencies of IMF-1, IMF-2, IMF-5, and IMF-6 and the TPs of IMF-1, IMF-3, IMF-4, and IMF-5 were identified as significant predictors. For a more targeted approach, we evaluated an MLR model using only eight IMF parameters (IMF-1_freq, IMF-2_freq, IMF-5_freq, IMF-6_freq, IMF-1_TP, IMF-3_TP, IMF-4_TP, and IMF-5_TP) that presented significant *p*-values (less than 0.05), and the model still presented excellent fit metrics (*R*^2^ = 0.879, MAE = 3.645, RMSE = 4.552) (Figure 6). This finding indicates that SSA effectively captures narrow-band changes in IMF parameters correlated with shifts in the BIS during emergence from GA, thereby demonstrating superior sensitivity for monitoring changes in the depth of anesthesia. The multiple linear regression equation derived from these eight significant parameters was used to predict BIS values over the 10-min period leading up to awakening, and the predicted values accurately reflected the actual changes in the measured BIS values.

Finally, Table 3 compares regression performance across four mode decomposition approaches (VMD, EWT, WMD, and SSA) using either a full 12-parameter model or a reduced model based on significant parameters [13,20]. Overall, SSA achieves a predictive performance comparable to the best-performing methods, as reflected by similar *R*^2^ values and low error metrics (MAE and RMSE), whereas EWT shows lower *R*^2^ and higher prediction errors. Notably, reducing the model to significant parameters preserves performance across methods, indicating that a limited subset of IMF-derived features contains most of the predictive information for BIS estimation. This comparative analysis shows that SSA-based decomposition provides competitive feature sets for BIS prediction while maintaining robust accuracy. In particular, CF2 and CF3 of SSA exhibited low-frequency noise-like fluctuations reflected in the wide IQR. These fluctuations have also been observed in the low-frequency region in VMD and EWT analyses and may reduce the correlation in multivariate regression analysis with BIS values [13,20]. In contrast, the previously reported WMD method successfully eliminated noise-like fluctuations in the low-frequency region [13]. As a result, as shown in the comparison in Table 3, the regression analysis of BIS values via WMD yielded the best coefficient of determination to date. There is room for improvement in data processing methods for IMFs that include the low-frequency region.

## 4. Discussion

Monitoring the depth of general anesthesia using EEG remains a complex challenge, largely due to variability in anesthetic agents and patient-specific factors such as age-related neurophysiological differences in pediatric and geriatric populations. Although EEG-based depth indices provided by commercially available monitors have proven useful [5,7], they are primarily calibrated for intravenous agents such as propofol and volatile anesthetics such as sevoflurane. This narrow optimization limits their generalizability to other anesthetic agents and patient groups. To improve depth-of-anesthesia monitoring, a more robust approach grounded in physiologically informed, scientifically validated algorithms is needed.

Quantitative analysis of these EEG spectral dynamics is essential in developing mathematical models that accurately capture the relationship between anesthetic pharmacodynamics and the depth of anesthesia. Such models could substantially enhance the precision and universality of depth-of-anesthesia assessments across diverse clinical scenarios. Frontal EEG signals recorded during GA exhibit characteristic changes in response to anesthetic-induced modulation of neurophysiological activity. Specifically, there is a marked suppression of higher-frequency *β* and *γ* activity—typically associated with alertness—while the power of slower-frequency components, such as δ and θ waves, increases, reflecting deeper hypnotic states [2,3,4]. Additionally, a pronounced rise in *α* activity, often associated with sleep spindles, is observed as anesthesia deepens.

To more effectively extract such EEG features, our recent study examined the use of mode decomposition techniques—EMD, VMD, and EWT—combined with the Hilbert transform, which offers a higher temporal resolution than conventional Fourier-based approaches [10,13,20]. While both VMD and EWT effectively capture frequency dynamics, they exhibit inconsistencies in assigning narrow-band components across successive epochs, limiting their reliability in continuous depth-of-anesthesia assessment. Moreover, wavelet mode decomposition (WMD), which we previously reported on as an application of EWT, can stabilize the central frequency and TP of the IMFs by using predefined frequency bands [13]. This method demonstrated a strong correlation with BIS values, particularly during the critical 10-min period from maintenance to emergence from anesthesia. Multiple regression analysis revealed that specific central frequencies and TP values of WMD-derived IMFs were significantly associated with changes in the depth of anesthesia. However, decomposing EEG signals into fixed frequency bands is conceptually equivalent to applying bandpass filters. Although this strategy is useful when the frequency bands of interest are well defined, it carries the inherent risk of missing characteristic changes that span multiple frequency bands, particularly when the relevant spectral features are not clearly established.

To address these limitations, we applied SSA for the first time to extract frequency features from EEG signals recorded during general anesthesia. EEG signals under general anesthesia exhibit time-varying, nonlinear, and non-stationary characteristics. SSA enables the decomposition of such complex signals while preserving their intrinsic statistical structure [25]. Unlike linear approaches such as the Fourier transform, SSA provides high-fidelity separation of trends, periodic components, and noise, making it a promising method for characterizing EEG dynamics under anesthesia.

In this study, we divided the EEG into six IMFs through SSA because it is common clinical practice to interpret human EEGs during general anesthesia using six frequency bands: δ, θ, α, low β, high β, and γ. However, our recent study on IMF number optimization using VMD showed that the optimal number of IMF decompositions varies from 2 to 6, depending on the anesthetic phase—from maintenance to emergence [26]. Dynamic optimization requires considerable computational processing time, making it difficult to incorporate into our current SSA-based mode decomposition algorithm. Therefore, we decomposed EEGs into six IMFs for comparison with previously reported VMD and EWT methods. To decompose the singular values obtained (128 in this case) into singular spectra using combinations of elementary matrices, we classified larger singular values with greater influence in more detail and gradually combined smaller singular values with less influence more broadly. This resulted in six IMFs with six combinations of elementary matrices: 0–1, 2–3, 4–6, 7–9, 10–19, and 20–127. This division method requires further refinement in future studies.

In contrast to the Fourier transform, which decomposes signals into frequency components to generate spectrograms, the combined application of mode decomposition and the Hilbert transform offers a more refined approach to feature extraction. This method enables the calculation of the IF and IA at each data point, producing high-resolution spectrograms that more accurately reflect the signal’s dynamic characteristics. In comparison, Fourier-based power spectral analysis—for instance, using 0.25 Hz frequency bins for a 128 Hz EEG sampling rate—requires at least 512 data points (equivalent to 4 s of EEG) and only achieves a temporal resolution of 2 s with a 50% window overlap. In this context, decomposing EEG signals into narrow-band intrinsic mode functions (IMFs) significantly improves the temporal resolution and enhances the granularity of time–frequency representations. The resulting Hilbert spectrogram allows for more detailed and precise analysis of non-stationary EEG signals.

The elementary components derived from SSA capture distinct rhythmic activities such as *α* (8–13 Hz) and *θ* (4–7 Hz) waves, which are modulated by anesthetic agents. These components change in proportion to the depth of anesthesia and can be used to extract quantitative, phase-preserving indicators of anesthetic depth. Trajectory matrices and singular vectors obtained via SSA effectively visualize and quantify dynamic changes in EEG across the different anesthetic phases—maintenance, transition, and emergence. This approach offers potential as a real-time analytic framework for assessing state transitions under general anesthesia. EEG decomposition via SSA, with the integration of deep learning and state-space modeling, holds promise in advancing precision anesthesia—individualized anesthetic management tailored to patient-specific neurophysiological responses. These innovations may enhance patient safety and reduce the risk of over-anesthesia.

In this study, we applied SSA-based mode decomposition to only 10 publicly available EEG cases with sevoflurane general anesthesia. Therefore, we cannot rule out potential overfitting in the multivariate linear regression analysis used to fit BIS values. Large-scale case studies are needed to establish the algorithm’s generalizability, which we consider a future challenge. Additionally, the limitations of this study are inherently linked to a fundamental and unresolved question: what precisely constitutes the depth of anesthesia [27]? Currently, commercially available monitors rely on indices such as the BIS and patient state index (PSi) as surrogate markers for anesthetic depth. However, the proprietary nature of the algorithms underlying these indices makes their validity difficult to verify through direct comparison. In this study, we examined the relationship between BIS values and parameters derived from the Hilbert spectra of IMFs obtained via mode decomposition. Although the algorithm used to calculate the BIS has not been fully disclosed, in this study, we demonstrated the potential for capturing changes in the frequency components of EEG signals during general anesthesia using singular spectrum analysis—a linear-algebra-based approach—and translating these findings into a technique for assessing the depth of anesthesia.

## 5. Conclusions

This study demonstrated that SSA combined with the Hilbert transform enables high-resolution, physiologically meaningful decomposition of EEG signals from patients under general anesthesia. SSA effectively extracted time-varying features associated with anesthetic depth transitions, offering superior temporal precision. Multiple regression models using SSA-derived features accurately predicted BIS values, highlighting the potential use of SSA in improving real-time anesthesia monitoring.

## Figures and Tables

**Table 1:** Comparison of mean IMF parameters (central frequency and total power) across three stages of general anesthesia.

	Maintenance		Transition			Emergence		
Parameter ^a^	Median ^b^	IQR	Median ^b^	%Change ^c^	IQR	Median ^b^	%Change ^c^	IQR
**CF1**	0.548 (*δ*)	0.722	0.523 (*δ*)	−4.6	0.857	0.42 †‡ (*δ*)	−23.4	0.569 †‡
**CF2**	6.076 (*θ*)	4.383	3.677 † (*δ*)	−39.5	6.058 †	2.517 †‡ (*δ*)	−58.6	3.685 †‡
CF3	6.073 (*θ*)	4.378	6.615 † (*θ*)	8.9	4.878 †	5.726 †‡ (*θ*)	−5.7	5.545 †
CF4	6.858 (*θ*)	3.652	7.322 † (*θ*)	6.8	4.254 †	7.21 † (*θ*)	5.1	5.463 †‡
**CF5**	7.610 (*θ*)	2.841	7.794 † (*θ*)	2.4	3.079	8.989 †‡ (*α*)	18.1	6.240 †‡
**CF6**	13.360 (*β*)	2.936	13.631 † (*β*)	2.0	3.510 †	17.358 †‡ (*β*)	29.9	6.019 †‡
**TP1**	9.222	11.459	7.779 †	−15.6	10.17 †	11.529 †‡	25.0	12.982 †‡
TP2	1.477	7.832	−0.13 †	−108.8	6.924 †	−0.458 †	−131.0	8.025 ‡
**TP3**	0.138	6.622	−1.692 †	−1326.1	5.693 †	−2.546 †‡	−1944.9	6.745 ‡
**TP4**	−1.767	7.078	−3.402 †	−92.5	5.992 †	−5.687 †‡	−221.8	5.8533 †
**TP5**	0.603	7.077	−0.874 †	−244.9	6.047 †	−3.434 †‡	−669.5	5.201 †‡
TP6	−2.560	7.716	−3.87 †	−51.2	6.196 †	−3.857 †	−50.7	5.157 †‡

Main characteristics of IMF parameters (central frequency and total power) decomposed by SSA. The 10-min data spanning from general anesthesia maintenance to emergence were divided into three periods of 3 min and 20 s each (maintenance, transition, and emergence phases). Mean values for each period were calculated and subjected to statistical analysis. ^a^ Significant parameters in the multivariate linear regression analysis are indicated in bold. ^b^ Calculated values were classified based on specific frequency bands and total power levels. ^c^ %change: Percentage change relative to the median value of the maintenance phase. The frequency spectrum was segmented into four bands: *δ* (<4 Hz), *θ* (4 to <8 Hz), *α* (8 to <13Hz), and *β* (13 to <30 Hz). CF: central frequency, TP: total power, IQR: interquartile range. Statistical comparisons were performed using the Kruskal–Wallis nonparametric test with Bonferroni correction. † *p* < 0.05 vs. the median of maintenance. ‡ *p* < 0.05 vs. the median of transition.

**Table 2:** Multiple linear regression analysis between the BIS values and parameters of the IMFs in the SSA.

**A. SSA with All 12 IMF Parameters**			
Determination factor (*R*^2^) MAE RMSE y-intercept		0.880 3.629 4.531 2.218	
Explanatory variables		Regression coefficient	*P* > |t|
Central frequency	IMF-1 IMF-2 IMF-3 IMF-4 IMF-5 IMF-6	−2.4592 0.5167 −0.1477 0.2697 1.1192 2.5711	0.035 * 0.001 * 0.362 0.201 0.000 * 0.000 *
Total power	IMF-1 IMF-2 IMF-3 IMF-4 IMF-5 IMF-6	0.2261 0.0395 −0.4074 −0.6025 −1.7897 −0.3360	0.005 * 0.777 0.025 * 0.004 * 0.000 * 0.085
**B. SSA with 8 Significant IMF Parameters**			
Determination factor (*R*^2^) MAE RMSE y-intercept		0.879 3.645 4.552 5.783	
Explanatory variables		Regression coefficient	*P* > |t|
Central frequency	IMF-1 IMF-2 IMF-5 IMF-6	−2.3968 0.4660 1.1003 2.4729	0.040 * 0.003 * 0.000 * 0.000 *
Total power	IMF-1 IMF-3 IMF-4 IMF-5	0.2262 −0.4358 −0.6260 −1.9634	0.003 * 0.011 * 0.003 * 0.000 *

The objective variable was the median BIS value from the 10 patients, and the explanatory variables were the median central frequencies and total powers of the IMFs in the SSA. The explanatory variables were (**A**) 12 IMF parameters or (**B**) 8 significant explanatory parameters. The EEG data were obtained from the last 10 min before emergence in 10 patients who received sevoflurane general anesthesia. MAE: mean absolute error; RMSE: root-mean-squared error; SSA: singular spectrum analysis; * *p* < 0.05.

**Table 3:** Multiple linear regression analysis between BIS values and IMF parameters: comparison of VMD, EWT, WMD and SSA methods.

**A. 12 IMF Parameter Model**				
	VMD	EWT	WMD	SSA
n of parameters	12	12	12	12
*R* ^2^	0.837	0.618	0.898	0.880
MAE	2.909	3.957	2.394	3.629
RMSE	3.751	5.33	3.128	4.531
**B. Significant IMF Parameter Model**				
	VMD	EWT	WMD	SSA
n of parameters	7	6	7	8
*R* ^2^	0.827	0.608	0.897	0.879
MAE	2.974	4.081	2.388	3.645
RMSE	3.865	5.394	3.148	4.552

IMF: intrinsic mode function, VMD: variational mode decomposition, EWT: empirical wavelet transform, WMD: wavelet mode decomposition, SSA: singular spectrum analysis, *R*^2^: determination factor, MAE: mean absolute error, RMSE: root-mean-squared error.

## Data Availability

The EEG dataset used herein is available on the author’s GitHub site (https://github.com/teijisw/EEG_DataSet, accessed on 13 December 2025) [13]. The programming codes used in the analysis in this paper are available as supplementary data.

## Supplementary Materials

The following supporting information can be downloaded at https://www.mdpi.com/article/10.3390/s26041212/s1. Supplementary file S1: File01_python_code for SVD (pdf). The Jupyter Notebook file of Python (ver. 3.8) code for the SSA. Supplementary file S2: File02_java_processing_code for SSA (text). Supplementary file S3: File03_EEG_data_10cases_EME10min.zip. Archive data file containing tab-separated values of the raw EEG data (microvolts) for 10 min before emergence from GA in ten patients. Supplementary files S4–S13: A video file showing SSA results of EEG data for the 30 min leading up to emergence in patients #1–#10. File04_Sev1_SSA.mp, patient #1. File05_Sev2_SSA.mp4, patient #2. File06_Sev3_SSA.mp4, patient #3. File07_Sev4_SSA.mp4, patient #4. File08_Sev5_SSA.mp4, patient #5. File09_Sev6_SSA.mp4, patient #6. File10_Sev7_SSA.mp4, patient #7. File11_Sev8_SSA.mp4, patient #8. File12_Sev9_SSA.mp4, patient #9. File13_Sev10_SSA.mp4, patient #10.

## Abbreviations

The following abbreviations are used in this manuscript:

BIS	bispectral index
DSA	density spectral arrays
EEG	electroencephalography
EMD	empirical mode decomposition
EMG_low_	absolute power derived from electromyography in the 70–110 Hz range
EWT	empirical wavelet transform
IMF	intrinsic mode function
IA	instantaneous amplitude
IF	instantaneous frequency
MLR	multiple linear regression
SEF_95_	95% spectral edge frequency
SSA	singular spectrum analysis
SVD	singular value decomposition
TP	total power
VMD	variational mode decomposition
WMD	wavelet mode decomposition

## References

[1] Yamada T., Obata Y., Sudo K., Kinoshita M., Naito Y., Sawa T. (2023). Changes in EEG frequency characteristics during sevoflurane general anesthesia: Feature extraction by variational mode decomposition. J. Clin. Monit. Comput..

[2] Obata Y., Yamada T., Akiyama K., Sawa T. (2023). Time-trend analysis of the center frequency of the intrinsic mode function from the Hilbert-Huang transform of electroencephalography during general anesthesia: A retrospective observational study. BMC Anesthesiol..

[3] Purdon P.L., Sampson A., Pavone K.J., Brown E.N. (2015). Clinical electroencephalography for anesthesiologists: Part I: Background and basic signatures. Anesthesiology.

[4] Supp G.G., Siegel M., Hipp J.F., Engel A.K. (2011). Cortical hypersynchrony predicts breakdown of sensory processing during loss of consciousness. Curr. Biol..

[5] Ching S., Cimenser A., Purdon P.L., Brown E.N., Kopell N.J. (2010). Thalamocortical model for a propofol-induced alpha-rhythm associated with loss of consciousness. Proc. Natl. Acad. Sci. USA.

[6] Flores F.J., Hartnack K.E., Fath A.B., Kim S.E., Wilson M.A., Brown E.N., Purdon P.L. (2017). Thalamocortical synchronization during induction and emergence from propofol-induced unconsciousness. Proc. Natl. Acad. Sci. USA.

[7] Roche D., Mahon P. (2021). Depth of Anesthesia Monitoring. Anesthesiol. Clin..

[8] Brook K., Agarwala A.V., Li F., Purdon P.L. (2024). Depth of anesthesia monitoring: An argument for its use for patient safety. Curr. Opin. Anesthesiol..

[9] Brook K., Ferraton M., Dreher L., Lambert D.H., Feldman J.M., Connor C.W. (2025). Electroencephalographic Depth-of-Anesthesia Monitoring. A A Pract..

[10] Nguyen-Ky T., Wen P., Li Y., Malan M. (2012). Measuring the hypnotic depth of anaesthesia based on the EEG signal using combined wavelet transform, eigenvector and normalisation techniques. Comput. Biol. Med..

[11] Huang N.E., Shen Z., Long S.R., Wu M.C., Shih H.H., Zheng Q., Yen N.-C., Tung C.C., Liu H.H. (1998). The empirical mode decomposition and the Hilbert spectrum for nonlinear and non-stationary time series analysis. Proc. Math. Phys. Eng. Sci..

[12] Dragomiretskiy K., Zosso D. (2014). Variational mode decomposition. IEEE Trans. Signal Process..

[13] Gilles J. (2013). Empirical wavelet transform. IEEE Trans. Signal Process..

[14] Yamochi S., Yamada T., Obata Y., Sudo K., Kinoshita M., Akiyama K., Sawa T. (2024). Wavelet transform-based mode decomposition for EEG signals under general anesthesia. PeerJ.

[15] D′Arcy J. Introducing SSA for Time Series Decomposition. https://www.kaggle.com/code/jdarcy/introducing-ssa-for-time-series-decomposition.

[16] Singular Spectrum Analysis. https://en.wikipedia.org/wiki/Singular_spectrum_analysis.

[17] Scikit-learn. Machine Learning in Python. https://scikit-learn.org/stable/.

[18] Kortelainen J., Vayrynen E. (2015). Assessing EEG slow wave activity during anesthesia using Hilbert-Huang Transform. Annu. Int. Conf. IEEE Eng. Med. Biol. Soc..

[19] Liu Q., Ma L., Fan S.Z., Abbod M.F., Ai Q., Chen K., Shieh J.S. (2018). Frontal EEG temporal and spectral dynamics similarity analysis between propofol and desflurane induced anesthesia using Hilbert-Huang transform. BioMed Res. Int..

[20] Li X., Li D., Liang Z., Voss L.J., Sleigh J.W. (2008). Analysis of depth of anesthesia with Hilbert-Huang spectral entropy. Clin. Neurophysiol..

[21] Shalbaf R., Behnam H., Sleigh J.W., Voss L.J. (2012). Using the Hilbert-Huang transform to measure the electroencephalographic effect of propofol. Physiol. Meas..

[22] Hayase K., Kainuma A., Akiyama K., Kinoshita M., Shibasaki M., Sawa T. (2021). Poincare plot area of gamma-band EEG as a measure of emergence from inhalational general anesthesia. Front. Physiol..

[23] Statsmodels. https://www.statsmodels.org/stable/index.html.

[24] Sawa T., Yamada T., Obata Y. (2022). Power spectrum and spectrogram of EEG analysis during general anesthesia: Python-based computer programming analysis. J. Clin. Monit. Comput..

[25] Hassani H. A Brief Introduction to Singular Spectrum Analysis. https://ssa.cf.ac.uk/ssa2010/a_brief_introduction_to_ssa.pdf.

[26] Kushimoto K., Obata Y., Yamada T., Kinoshita M., Akiyama K., Sawa T. (2024). Variational Mode Decomposition Analysis of Electroencephalograms during General Anesthesia: Using the Grey Wolf Optimizer to Determine Hyperparameters. Sensors.

[27] Sleigh J.W. (2011). Depth of anesthesia: Perhaps the patient isn’t a submarine. Anesthesiology.

