# Supplementary material for: Assessment of Anesthetic Depth Through EEG Mode Decomposition Using Singular Spectrum Analysis
Source: Sensors (Basel). 2026 Feb 12;26(4):1212. doi: 10.3390/s26041212 (PMC12943922; doi:10.3390/s26041212)

SSA_240810_maintenance

December 24, 2025

1 Spectral Analysis: Singular Spectrum Analysis #240810, by Python 3.8

1.1 Aug 10, 2024 & Dec 23, 2025 by Teiji Sawa, MD, PhD, Kyoto Prefectural University of Medicine

Reference: JORDAN D'ARCY, Introducing SSA for Time Series Decomposition. https://www.kaggle.com/code/jdarcy/introducing-ssa-for-time-series-decomposition

2 #Spectral analysis of EEG, L = 128, maintenance

In this section, we will perform spectrum analysis by Python using EEG Analyzer and digital EEG data file eeg_bis.tsv. The influence of window functions, the application of the multitaper method, and the development into spectrograms will be shown.

3 EEG analysis

[1]:
```
import numpy as np
import pandas as pd
import matplotlib.pyplot as plt
from scipy.fftpack import fft
from scipy import signal as sig #Signal function import from SciPy package
from numpy import dot, multiply, diag, power
from numpy.linalg import inv, eig, pinv
from numpy import pi, exp, log
from scipy.linalg import svd, svdvals, diagsvd

# Fiddle with figure settings here:
plt.rcParams['figure.figsize'] = (10,8)
plt.rcParams['font.size'] = 14
plt.rcParams['image.cmap'] = 'plasma'
plt.rcParams['axes.linewidth'] = 2

from cycler import cycler
cols = plt.get_cmap('tab10').colors
plt.rcParams['axes.prop_cycle'] = cycler(color=cols)
```

```python
from IPython.display import set_matplotlib_formats
#set_matplotlib_formats('svg')
```

First, the EEG data from the digital EEG data file eeg_bis_10_20_2.tsv acquired from the BIS monitor through the EEG Analyzer are imported into the Pandas DataFrame df_tsv.

[2]:
```python
#df_tsv = pd.read_table('./PRO_Case01_20210319_EME10.tsv')
df_tsv = pd.read_table('./Github/EEG_DataSet/general_anesth_sevoflurane/
 ↪EME10min/Sev_Case_01_EME10min.tsv')
```

Let's display the first five items of the imported data set.

[3]:
```python
df_tsv.head(5)
```

[3]:
```
     Ch      Time  ch[0]  ch[1]  ch[2]  ch[3]  ch[4]  ch[5]  ch[6]  ch[7]  \
0  ch1:  12:22:40  21.95  29.75  30.05  23.90  14.05   7.00  -0.05 -10.50
1  ch1:  12:22:40  -6.05  -9.30 -12.30 -13.00 -10.95  -3.80   4.50  12.25
2  ch1:  12:22:40  11.10  21.10  23.90  21.15  15.40   6.75  -5.60 -19.45
3  ch1:  12:22:40  -0.60   4.20   5.15   0.30  -8.90 -16.50 -20.95 -21.50
4  ch1:  12:22:40  -2.50  -6.90  -6.80   0.05   8.70  15.75  22.25  24.90

   ch[8]  ch[9]  ch[10]  ch[11]  ch[12]  ch[13]  ch[14]  ch[15]
0 -18.45 -22.55  -24.75  -24.80  -23.35  -21.05  -15.05   -7.80
1  21.25  27.05   20.80    1.90  -21.00  -32.15  -23.50   -5.05
2 -26.35 -21.45  -14.05  -13.70  -18.30  -20.10  -15.90   -7.75
3 -17.60  -9.45    1.50   11.20   16.95   18.95   14.65    5.45
4  21.55  13.40    1.35   -8.45   -7.65    2.45   15.50   27.00
```

Let's display the first five items of the imported data set.

[4]:
```python
df_tsv.tail(5)
```

[4]:
```
        Ch      Time  ch[0]  ch[1]  ch[2]  ch[3]  ch[4]  ch[5]  ch[6]  ch[7]  \
4795  ch1:  12:32:54  -0.40   3.30   5.65   1.70    0.9   9.05   9.70   4.70
4796  ch1:  12:32:54   8.70  -0.25  -1.25   1.30    5.4   9.30   6.50   2.40
4797  ch1:  12:32:54   5.75   7.00   7.95  10.05   16.7  16.60   9.00   9.25
4798  ch1:  12:32:54   8.20   8.75  13.45  17.70   15.7  18.25  16.35  11.70
4799  ch1:  12:32:54  25.35  25.10  11.00   7.85   11.0  19.10  19.60  12.80

      ch[8]  ch[9]  ch[10]  ch[11]  ch[12]  ch[13]  ch[14]  ch[15]
4795   4.30   1.80   -2.15   -2.45    2.70    6.50   13.65   17.30
4796  10.45  14.00   10.75    9.35    4.85    3.70   12.30   12.00
4797  10.05   9.20   14.00   15.45   10.05   12.15   10.65   10.90
4798   4.65   7.60   17.95   20.40   15.60   21.00   19.90   20.10
4799  11.30  13.95   15.55   16.85   17.10   20.70   21.00   12.25
```

Create a new Pandas DataFrame df_eeg and set the column names as "Time" and "eeg".

[5]:
```
cols = ['Time', 'eeg']
df_eeg = pd.DataFrame(data=None, index=[], columns=cols)
```

Of the df_tsv (line 109611), copy the voltage µV data corresponding to the status under deep sevoflurane anesthesia (line 97,000-99,048) to df_eeg.

[6]:
```
#for row in range(0, 4682):
    # Import only 1024 lines of data. Since 8 lines / second, 128 seconds = 2␣
↪minutes 8 seconds
    # Number of data = 16,384
    # By playing with the range here, you can change which brain waves you can␣
↪take out.

#    t_tmp = df_tsv.iat[row,1]
#    for column in range(2, 18):
#              eeg_tmp = df_tsv.iat[row, column]
#              data_tmp = pd.Series( [t_tmp, eeg_tmp], index=df_eeg.columns )
#              df_eeg = df_eeg.append(data_tmp, ignore_index=True)

cols = ['Time', 'eeg']
temp_data = []  # Initialization

for row in range(len(df_tsv)):
#for row in range(0, 2048):
#for row in range(0, 4682):
#for row in range(0, 4696):
    t_tmp = df_tsv.iat[row, 1]
    for column in range(2, 18):
        eeg_tmp = df_tsv.iat[row, column]
        data_tmp = [t_tmp, eeg_tmp]  # add data as list
        temp_data.append(data_tmp)

# temp_data Making DataFrame from list
df_eeg = pd.DataFrame(temp_data, columns=cols)
```

Let's confirm and display 1,024 rows x 16 = 16,383 µV data (for 128 seconds) captured in df_eeg.

[7]:
```
df_eeg
```

[7]:

	Time	eeg
0	12:22:40	21.95
1	12:22:40	29.75
2	12:22:40	30.05
3	12:22:40	23.90
4	12:22:40	14.05
…	…	…
76795	12:32:54	16.85
76796	12:32:54	17.10

```
76797  12:32:54  20.70
76798  12:32:54  21.00
76799  12:32:54  12.25

[76800 rows x 2 columns]
```

Save the data imported to df_eeg as a csv file.

[8]:
```
df_eeg.to_csv("Sev_case01_EME10.csv")
```

Transfer the df_eeg time and µV data to the new Python arrays Time and eeg, respectively.

[9]:
```
#Place brain wave data in the data frame
time = df_eeg['Time'].values
eeg = df_eeg['eeg'].values
```

Display the data captured in the array eeg using Matplotlib function plot().

[10]:
```
#Time-series graph display of EEG data
fig, ax = plt.subplots(figsize=(18,6))
ax.plot(eeg)
#plt.savefig('./Fig_1_Sev_Case01.svg')
```

[10]:
```
[<matplotlib.lines.Line2D at 0x1645daca0>]
```

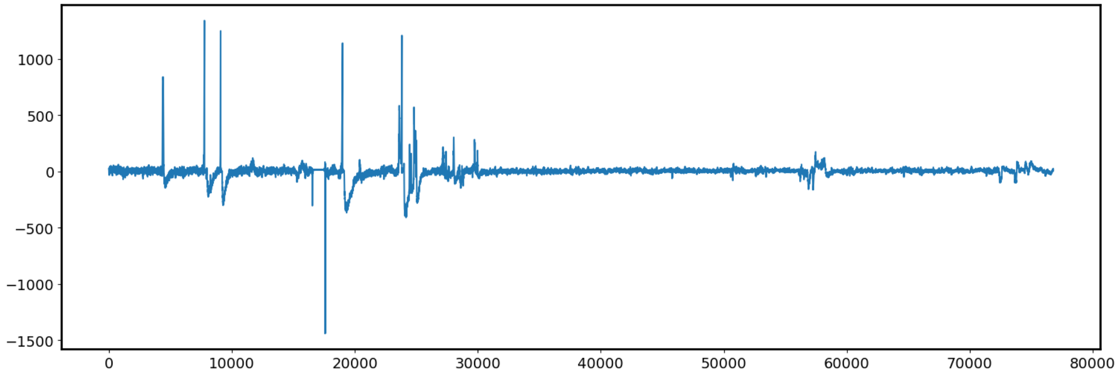

4 Data preprocessing

Let's calculate the mean and standard deviation of eeg.

[11]:
```
m=np.mean(eeg)
sd=np.std(eeg)
m, sd
```

[11]:
```
(-0.7308678385416665, 68.60588143250526)
```

Count the number of null (NA) data.

[12]:
```
df_eeg['eeg'].isnull().sum()
```

[12]:
```
0
```

Outlier removal: Change the data that exceeds 3 times the standard deviation from the mean value to None.

[13]:
```
#for i in range(len(eeg)):
#    if  df_eeg['eeg'][i] > m+3*sd:
#      df_eeg['eeg'][i] = None

df_eeg.loc[df_eeg['eeg'] > m + 3 * sd, 'eeg'] = None
```

[14]:
```
#for i in range(len(eeg)):
#    if  df_eeg['eeg'][i] < m-3*sd:
#      df_eeg['eeg'][i] = None

df_eeg.loc[df_eeg['eeg'] < m - 3 * sd, 'eeg'] = None
```

After removing the outliers, count the number of null (NA) data again.

[15]:
```
df_eeg['eeg'].isnull().sum()
```

[15]:
```
1767
```

Make a copy of df_eeg and perfrom spline interpolation.

[16]:
```
df_eeg_copy = df_eeg.copy()
df_eeg_copy.interpolate(inplace=True)
```

Count the number of null (NA) data in the copy of df_eeg.

[17]:
```
df_eeg_copy['eeg'].isnull().sum()
```

[17]:
```
0
```

Save the data with spline completion to a csv file.

[18]:
```
#df_eeg.to_csv("eeg_linealized_Sev_Case01.csv")
```

Copy the eeg data to the array eeg_ip.

[19]:
```
eeg_ip = df_eeg_copy['eeg'].values
```

Plot and display eeg data.

[20]:
```
#Time-series graph display of EEG data that complements and corrects missing␣
 ↪values
fig, ax = plt.subplots(figsize=(18,6))
ax.plot(eeg_ip)
```

```
#plt.savefig('Fig_2_Sev_Case01.svg')
```

[20]: [<matplotlib.lines.Line2D at 0x1647ad6d0>]

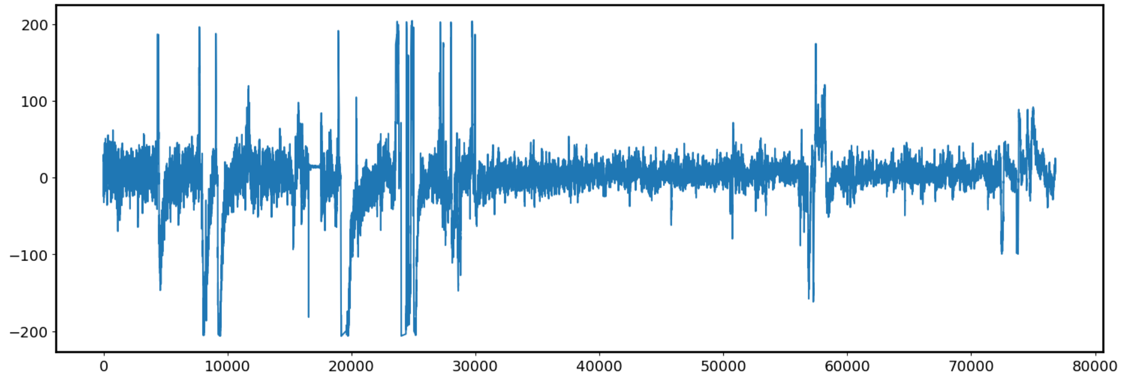

From the eeg data, 512 cases (4 seconds, eeg_512) and 8,192 cases (64 seconds, eeg_8192) are extracted.

[21]:
```
t1 = np.linspace(0, 3, 384)
```

[22]:
```
t1_int = np.linspace(0, 384, 384, dtype = 'int')
```

[23]:
```
t = np.linspace(0, 1.0, 128)
```

[24]:
```
t2 = np.linspace(0, 128, 128, dtype = 'int')
```

[25]:
```
t[127]
```

[25]: 1.0

[26]:
```
t1_int[383]
```

[26]: 384

[27]:
```
t2[127]
```

[27]: 128

[28]:
```
dt = t[2] - t[1] # sampling period
dt
```

[28]: 0.007874015748031496

[29]:
```
N=383
L=128
K = N - L + 1
```

```
K
```

[29]: 256

[30]:
```
eeg_384 = eeg_ip[0:0+384]
#time_512 = time[13000:13512]
eeg_8192 = eeg_ip[0:8192]
time_8192 = time[0:8192]

#maintenance

for i in range(0, 256):
    locals()[f"eeg_128_{i}"] = np.array([eeg_ip[0+i:0+i+128]])
```

[31]:
```
eeg_128_0
```

[31]:
```
array([[ 21.95,  29.75,  30.05,  23.9 ,  14.05,   7.  ,  -0.05, -10.5 ,
        -18.45, -22.55, -24.75, -24.8 , -23.35, -21.05, -15.05,  -7.8 ,
         -6.05,  -9.3 , -12.3 , -13.  , -10.95,  -3.8 ,   4.5 ,  12.25,
         21.25,  27.05,  20.8 ,   1.9 , -21.  , -32.15, -23.5 ,  -5.05,
         11.1 ,  21.1 ,  23.9 ,  21.15,  15.4 ,   6.75,  -5.6 , -19.45,
        -26.35, -21.45, -14.05, -13.7 , -18.3 , -20.1 , -15.9 ,  -7.75,
         -0.6 ,   4.2 ,   5.15,   0.3 ,  -8.9 , -16.5 , -20.95, -21.5 ,
        -17.6 ,  -9.45,   1.5 ,  11.2 ,  16.95,  18.95,  14.65,   5.45,
         -2.5 ,  -6.9 ,  -6.8 ,   0.05,   8.7 ,  15.75,  22.25,  24.9 ,
         21.55,  13.4 ,   1.35,  -8.45,  -7.65,   2.45,  15.5 ,  27.  ,
         35.4 ,  42.25,  44.65,  40.95,  32.45,  20.8 ,  12.2 ,  10.3 ,
          9.5 ,  11.  ,  18.75,  28.7 ,  36.55,  41.05,  40.1 ,  38.15,
         36.  ,  27.15,  16.85,  12.65,  13.  ,  15.55,  20.  ,  25.8 ,
         36.2 ,  45.55,  42.95,  30.55,  19.75,  16.1 ,  19.4 ,  26.95,
         35.7 ,  43.6 ,  44.1 ,  34.85,  21.85,  10.2 ,   2.25,  -1.2 ,
         -1.  ,   2.85,   7.5 ,   9.65,  11.15,  13.5 ,  14.8 ,  14.2 ]])
```

[32]:
```
#Time-series graph display of EEG data that complements and corrects missing␣
↪values
ax = []
plt.clf()
ax_count = 1

fig = plt.figure(figsize=(18,3))
plt.ylim(-100, 100)
plt.plot(eeg_384[0:384], linewidth =0.5)
plt.savefig('Sev_case01_eeg_192.svg')
```

```
<Figure size 1000x800 with 0 Axes>
```

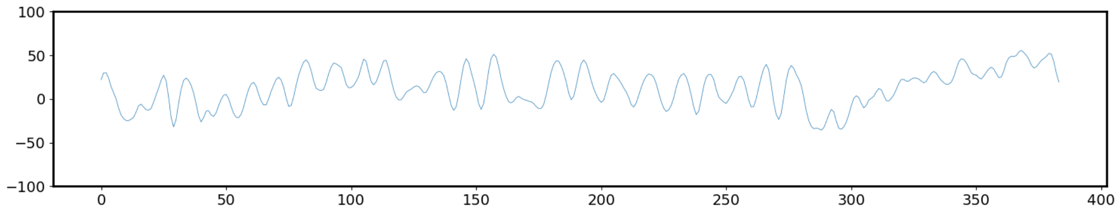

[33]:
```
#Time-series graph display of EEG data that complements and corrects missing␣
↪values
ax = []
plt.clf()
ax_count = 1

fig = plt.figure(figsize=(18,3))
plt.ylim(-100, 100)
plt.plot(eeg_384[0:256], linewidth =0.5)
#plt.savefig('Fig_4_PRO_Case01.svg')
```

[33]: [<matplotlib.lines.Line2D at 0x164890fd0>]

<Figure size 1000x800 with 0 Axes>

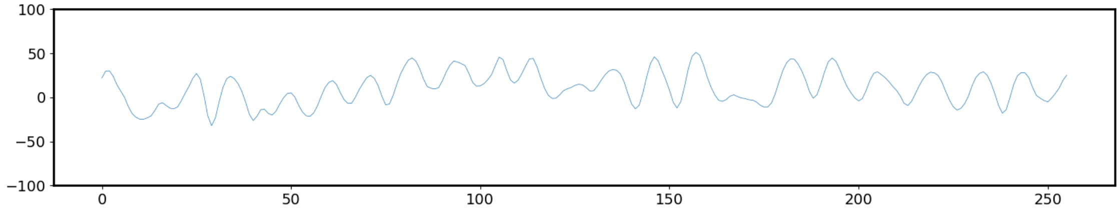

[34]:
```
df_eeg_384 = pd.DataFrame(eeg_384)
df_eeg_384.to_csv("df_eeg_384.csv")
```

[35]:
```
eeg_128 = eeg_128_0

for i in range(1, 256):
    if 'eeg_128_{}'.format(i) in locals():
        eeg_128 = np.concatenate([eeg_128, locals()[f'eeg_128_{i}']])

eeg_128
```

[35]:
```
array([[21.95, 29.75, 30.05, …, 13.5 , 14.8 , 14.2 ],
       [29.75, 30.05, 23.9 , …, 14.8 , 14.2 , 11.1 ],
       [30.05, 23.9 , 14.05, …, 14.2 , 11.1 ,  7.15],
```

```
…,
[10.5 , 19.2 , 25.15, …, 48.65, 52.05, 51.45],
[19.2 , 25.15, 25.95, …, 52.05, 51.45, 42.7 ],
[25.15, 25.95, 21.55, …, 51.45, 42.7 , 29.6 ]])
```

Let's display of eeg_512

[36]:
```
# combine signals and make data matrix
D = eeg_128.T

# create DMD input-output matrices
X = D[:,:-1]
Y = D[:,1:]
t3 = np.arange(0, K)
```

[37]:
```
ax = plt.matshow(D)
plt.xlabel("$L$-Lagged Vectors")
plt.ylabel("$K$-Lagged Vectors")
plt.colorbar(ax.colorbar, fraction=0.025)
ax.colorbar.set_label("$F(t)$")
plt.title("The Trajectory Matrix for the EEG Time Series");
plt.savefig('Sev_case01_Trajectory_1_10min.svg')
```

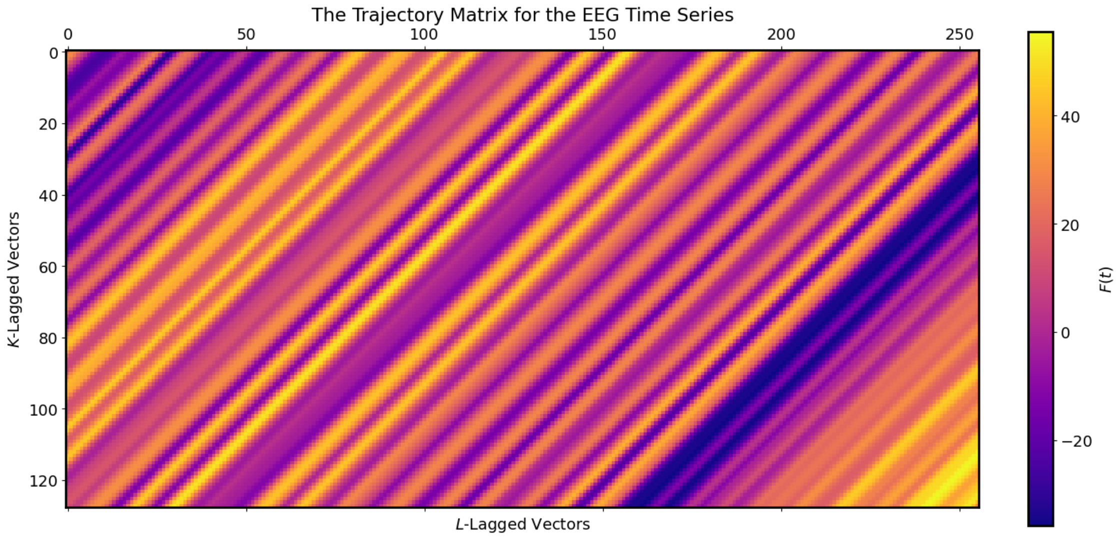

[38]:
```
len(t3)
```

[38]: 256

[39]:
```
t3
```

[39]:

```
array([  0,   1,   2,   3,   4,   5,   6,   7,   8,   9,  10,  11,  12,
        13,  14,  15,  16,  17,  18,  19,  20,  21,  22,  23,  24,  25,
        26,  27,  28,  29,  30,  31,  32,  33,  34,  35,  36,  37,  38,
        39,  40,  41,  42,  43,  44,  45,  46,  47,  48,  49,  50,  51,
        52,  53,  54,  55,  56,  57,  58,  59,  60,  61,  62,  63,  64,
        65,  66,  67,  68,  69,  70,  71,  72,  73,  74,  75,  76,  77,
        78,  79,  80,  81,  82,  83,  84,  85,  86,  87,  88,  89,  90,
        91,  92,  93,  94,  95,  96,  97,  98,  99, 100, 101, 102, 103,
       104, 105, 106, 107, 108, 109, 110, 111, 112, 113, 114, 115, 116,
       117, 118, 119, 120, 121, 122, 123, 124, 125, 126, 127, 128, 129,
       130, 131, 132, 133, 134, 135, 136, 137, 138, 139, 140, 141, 142,
       143, 144, 145, 146, 147, 148, 149, 150, 151, 152, 153, 154, 155,
       156, 157, 158, 159, 160, 161, 162, 163, 164, 165, 166, 167, 168,
       169, 170, 171, 172, 173, 174, 175, 176, 177, 178, 179, 180, 181,
       182, 183, 184, 185, 186, 187, 188, 189, 190, 191, 192, 193, 194,
       195, 196, 197, 198, 199, 200, 201, 202, 203, 204, 205, 206, 207,
       208, 209, 210, 211, 212, 213, 214, 215, 216, 217, 218, 219, 220,
       221, 222, 223, 224, 225, 226, 227, 228, 229, 230, 231, 232, 233,
       234, 235, 236, 237, 238, 239, 240, 241, 242, 243, 244, 245, 246,
       247, 248, 249, 250, 251, 252, 253, 254, 255])
```

[40]:

```
ax = []
plt.clf()
ax_count = 1
max_plot_num = 20
indx = 10
```

```
<Figure size 1000x800 with 0 Axes>
```

[41]:

```
U,Sig,Vh = svd(X)

sigma_sq = (Sig ** 2)
sigma_sumsq = (Sig ** 2).sum()
relative_contrib = (Sig ** 2)/ sigma_sumsq * 100
cumlative_contrib = (Sig ** 2).cumsum() / sigma_sumsq * 100
sigma_sq = (Sig ** 2)
sigma_sumsq = (Sig ** 2).sum()
relative_contrib = (Sig ** 2)/ sigma_sumsq * 100
cumlative_contrib = (Sig ** 2).cumsum() / sigma_sumsq * 100
```

[42]:

```
fig, ax = plt.subplots(1, 2, figsize=(14,5))
ax[0].plot(Sig**2 / sigma_sumsq * 100, lw=2.5)
ax[0].set_xlim(0,60)
ax[0].set_title("Relative Contribution of $\mathbf{X}_i$ to Trajectory Matrix")
ax[0].set_xlabel("$i$")
ax[0].set_ylabel("Contribution (%)")
ax[1].plot((Sig**2).cumsum() / sigma_sumsq * 100, lw=2.5)
ax[1].set_xlim(0,60)
```

```
ax[1].set_title("Cumulative Contribution of $\mathbf{X}_i$ to Trajectory␣
↪Matrix")
ax[1].set_xlabel("$i$")
ax[1].set_ylabel("Contribution (%)");
plt.savefig('Sev_case01_Contribution.svg')
```

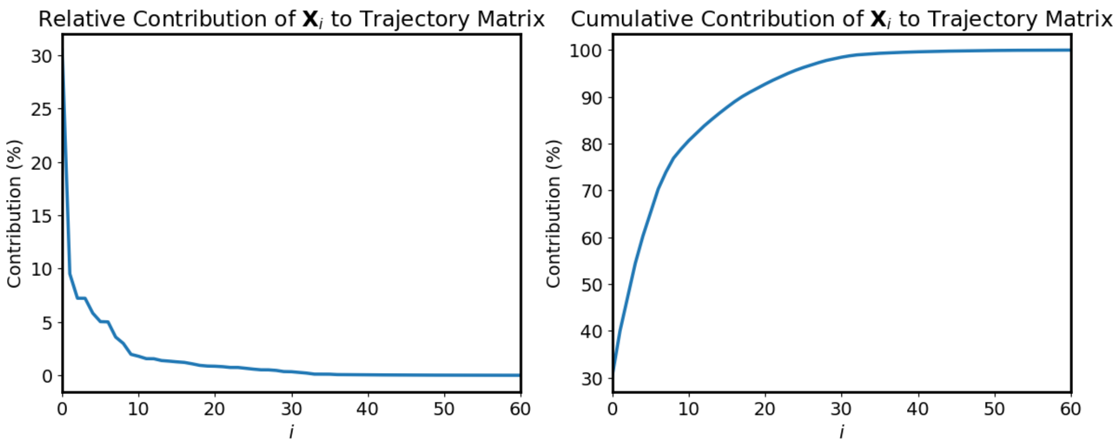

[43]:
```
contrib1 = Sig**2 / sigma_sumsq * 100
pd_contrib1 = pd.DataFrame(contrib1)
pd_contrib1.to_csv("Sev_case01_contrib1.csv")
```

[44]:
```
contrib2 = (Sig**2).cumsum() / sigma_sumsq * 100
pd_contrib2 = pd.DataFrame(contrib2)
pd_contrib2.to_csv("Sev_case01_contrib2.csv")
```

[45]:
```
#CCC
# A simple little 2D matrix plotter, excluding x and y labels.
def plot_2d(m, title=""):
    plt.imshow(m)
    plt.xticks([])
    plt.yticks([])
    plt.title(title)
```

[46]:
```
ax = []
plt.clf()
ax_count = 1
max_plot_num = 3

fig = plt.figure(figsize=(18, 8))
fig.subplots_adjust(hspace=1)

ax.append(fig.add_subplot((max_plot_num), 1, ax_count))
```

```
ax[-1].set_xlabel('t')
ax[-1].set_ylabel('y')
ax[-1].set_title('SVD')
ax[-1].scatter(range(0, len(Sig)), Sig, label="SVD")
ax_count += 1

ax.append(fig.add_subplot((max_plot_num), 1, ax_count))
ax[-1].set_xlabel('t')
ax[-1].set_ylabel('y')
ax[-1].set_ylim(0, 100)
ax[-1].set_title('SVD')
ax[-1].scatter(range(0, len(cumlative_contrib)), relative_contrib, label="SVD")
ax_count += 1

ax.append(fig.add_subplot((max_plot_num), 1, ax_count))
ax[-1].set_xlabel('t')
ax[-1].set_ylabel('y')
ax[-1].set_ylim(0, 100)
ax[-1].set_title('SVD')
ax[-1].scatter(range(0, len(cumlative_contrib)), cumlative_contrib, label="SVD")
ax_count += 1

plt.show()
```

```
<Figure size 1000x800 with 0 Axes>
```

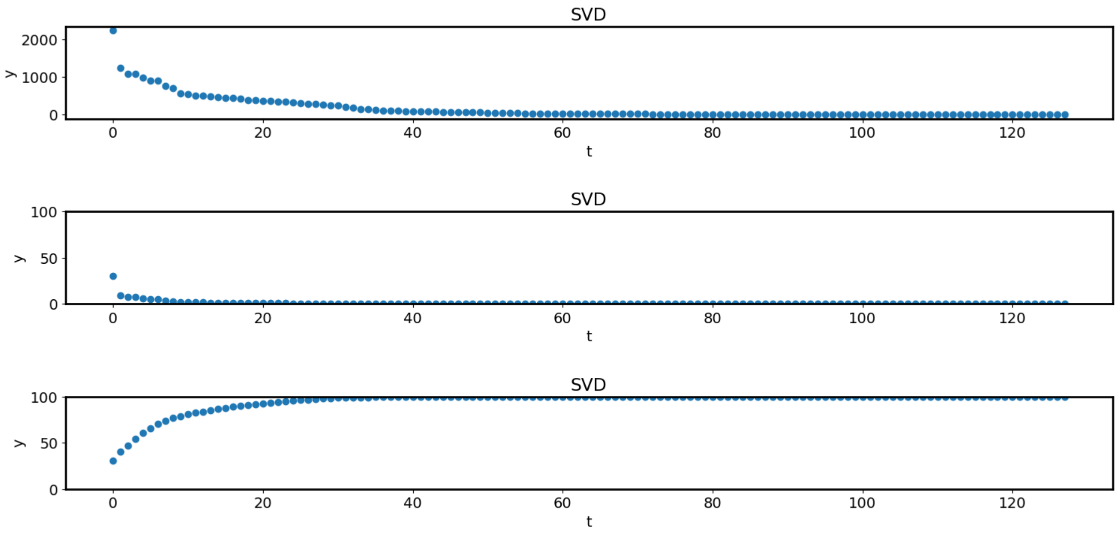

[47]:
```
# rank-3 truncation
r =124
U2 = U[:,:r]
```

```
Sig2 = diag(Sig)[:r,:r]
Vh2 = Vh.conj().T[:,:r]

# build A tilde
Atil = dot(dot(dot(U2.conj().T, Y), Vh2), inv(Sig2))
mu,W = eig(Atil)

# build DMD modes
Phi = dot(dot(dot(Y, Vh2), inv(Sig2)), W)

# compute time evolution
#b = dot(pinv(Phi), X[:,0])
#Psi = np.zeros([r, len(t)], dtype='complex')
#for i,_t in enumerate(t):
#    Psi[:,i] = multiply(power(mu, _t/dt), b)

#
b = dot(pinv(Phi), X[:,0])
Psi = np.zeros([r, 256], dtype="complex")
for idx, mu_elem in enumerate(mu):
    for _k in t3:
        Psi[idx, _k] = exp(log(mu_elem) / dt * _k * dt) * b[idx]
```

[48]:

```
#CCC
d = np.linalg.matrix_rank(X) # The intrinsic dimensionality of the trajectory␣
 ↪space.

# For those interested in how to code up an SVD calculation, Numerical Recipes␣
 ↪in Fortran 77
# has you covered: http://www.aip.de/groups/soe/local/numres/bookfpdf/f2-6.pdf
# Thankfully, we'll leave the actual SVD calculation to NumPy.
#U_c, Sigma_c, V_c = svd(X)
#U2,Sig2,Vh2 = svd(X)

Vh_t = Vh.T # Note: the SVD routine returns V^T, not V, so I'll tranpose it␣
 ↪back here. This may seem pointless,
# but I'll treat the Python representation of V consistently with the␣
 ↪mathematical notation in this notebook.

# Calculate the elementary matrices of X, storing them in a multidimensional␣
 ↪NumPy array.
# This requires calculating sigma_i * U_i * (V_i)^T for each i, or sigma_i *␣
 ↪outer_product(U_i, V_i).
# Note that Sigma is a 1D array of singular values, instead of the full L x K␣
 ↪diagonal matrix.
```

```
#X_elem = np.array( [Sigma_c[i] * np.outer(U_c[:,i], V_c[:,i]) for i in␣
↪range(0,d)] )
X_elem = np.array( [Sig[i] * np.outer(U[:,i], Vh_t[:,i]) for i in range(0,d)] )


# Quick sanity check: the sum of all elementary matrices in X_elm should be␣
↪equal to X, to within a
# *very small* tolerance:
if not np.allclose(X, X_elem.sum(axis=0), atol=1e-10):
    print("WARNING: The sum of X's elementary matrices is not equal to X!")
```

[49]:
```
d
```

[49]: 128

[50]:
```
#CCC
def Hankelise(X):
    """
    Hankelises the matrix X, returning H(X).
    """
    L, K = X.shape
    transpose = False
    if L > K:
        # The Hankelisation below only works for matrices where L < K.
        # To Hankelise a L > K matrix, first swap L and K and tranpose X.
        # Set flag for HX to be transposed before returning.
        X = X.T
        L, K = K, L
        transpose = True

    HX = np.zeros((L,K))

    # I know this isn't very efficient...
    for m in range(L):
        for n in range(K):
            s = m+n
            if 0 <= s <= L-1:
                for l in range(0,s+1):
                    HX[m,n] += 1/(s+1)*X[l, s-l]
            elif L <= s <= K-1:
                for l in range(0,L-1):
                    HX[m,n] += 1/(L-1)*X[l, s-l]
            elif K <= s <= K+L-2:
                for l in range(s-K+1,L):
                    HX[m,n] += 1/(K+L-s-1)*X[l, s-l]
    if transpose:
        return HX.T
```

```
    else:
        return HX
```

[51]:
```
n = min(d, r)
for j in range(0,20):
    plt.subplot(4,5,j+1)
    title = r"$\tilde{\mathbf{X}}_{" + str(j) + "}$"
    plot_2d(Hankelise(X_elem[j]), title)
plt.tight_layout()
plt.savefig('Sev_case01_Hankelise.svg')
```

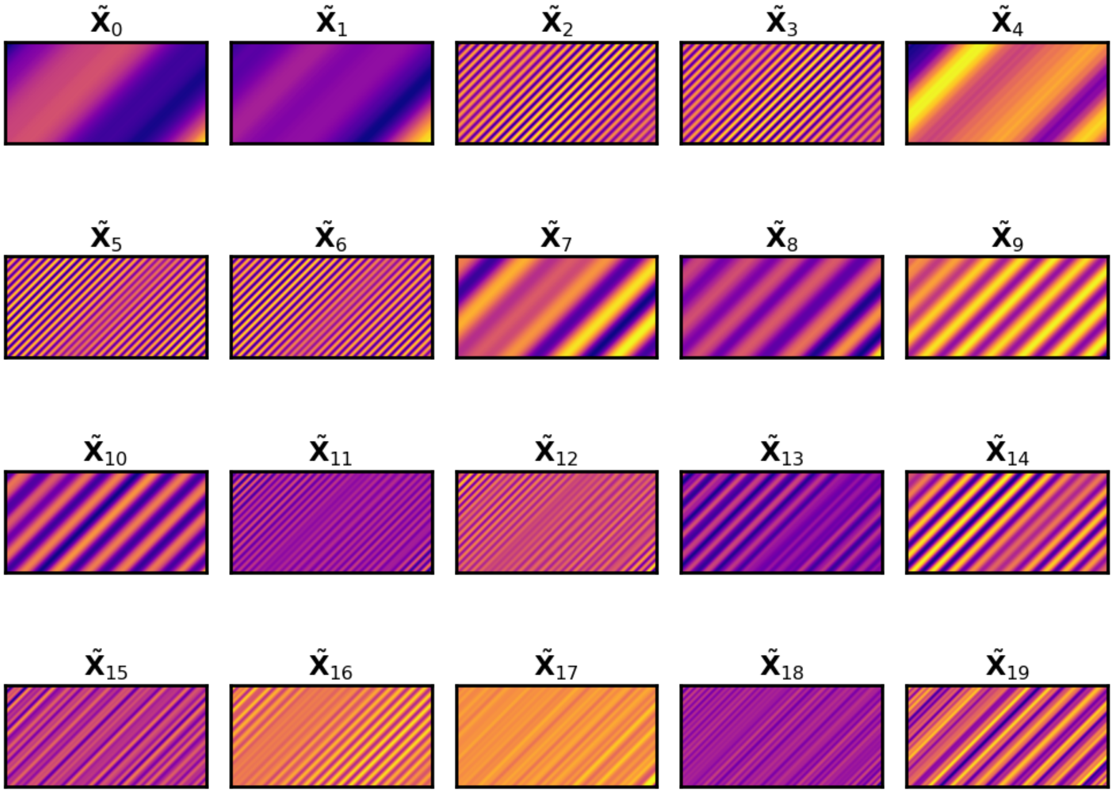

[52]:
```
def circle(r=1):
    x,y = [],[]
    for _x in np.linspace(-180,180,360):
        x.append(np.sin(np.radians(_x)))
        y.append(np.cos(np.radians(_x)))
    return x,y

c_x,c_y = circle(r=1)

fig, ax = plt.subplots(figsize=(8,8))
```

```
ax.plot(c_x, c_y, c="k", linestyle="dashed")
ax.scatter(np.real(mu[0]), np.imag(mu[0]), label="1st")
ax.scatter(np.real(mu[1]), np.imag(mu[1]), label="2nd")
ax.scatter(np.real(mu[2]), np.imag(mu[2]), label="3rd")
ax.scatter(np.real(mu[3]), np.imag(mu[3]), label="4th")
ax.scatter(np.real(mu[4]), np.imag(mu[4]), label="5th")
ax.scatter(np.real(mu[5]), np.imag(mu[5]), label="6th")
ax.scatter(np.real(mu[6]), np.imag(mu[6]), label="7th")
ax.scatter(np.real(mu[7]), np.imag(mu[7]), label="8th")
ax.scatter(np.real(mu[8]), np.imag(mu[8]), label="9th")
ax.scatter(np.real(mu[9]), np.imag(mu[9]), label="10th")

ax.scatter(np.real(mu[10]), np.imag(mu[10]), label="11th")
ax.scatter(np.real(mu[11]), np.imag(mu[11]), label="12th")
ax.scatter(np.real(mu[12]), np.imag(mu[12]), label="13th")
ax.scatter(np.real(mu[13]), np.imag(mu[13]), label="14th")
ax.scatter(np.real(mu[14]), np.imag(mu[14]), label="15th")
ax.scatter(np.real(mu[15]), np.imag(mu[15]), label="16th")
ax.scatter(np.real(mu[16]), np.imag(mu[16]), label="17th")
ax.scatter(np.real(mu[17]), np.imag(mu[17]), label="18th")
ax.scatter(np.real(mu[18]), np.imag(mu[18]), label="19th")
ax.scatter(np.real(mu[19]), np.imag(mu[19]), label="20th")

ax.set_aspect("equal")
ax.set_xlabel(r"$\it{Re}\,\mu$")
ax.set_ylabel(r"$\it{Im}\,\mu$")
ax.legend()

plt.savefig('Sev_case01_circle.svg')
```

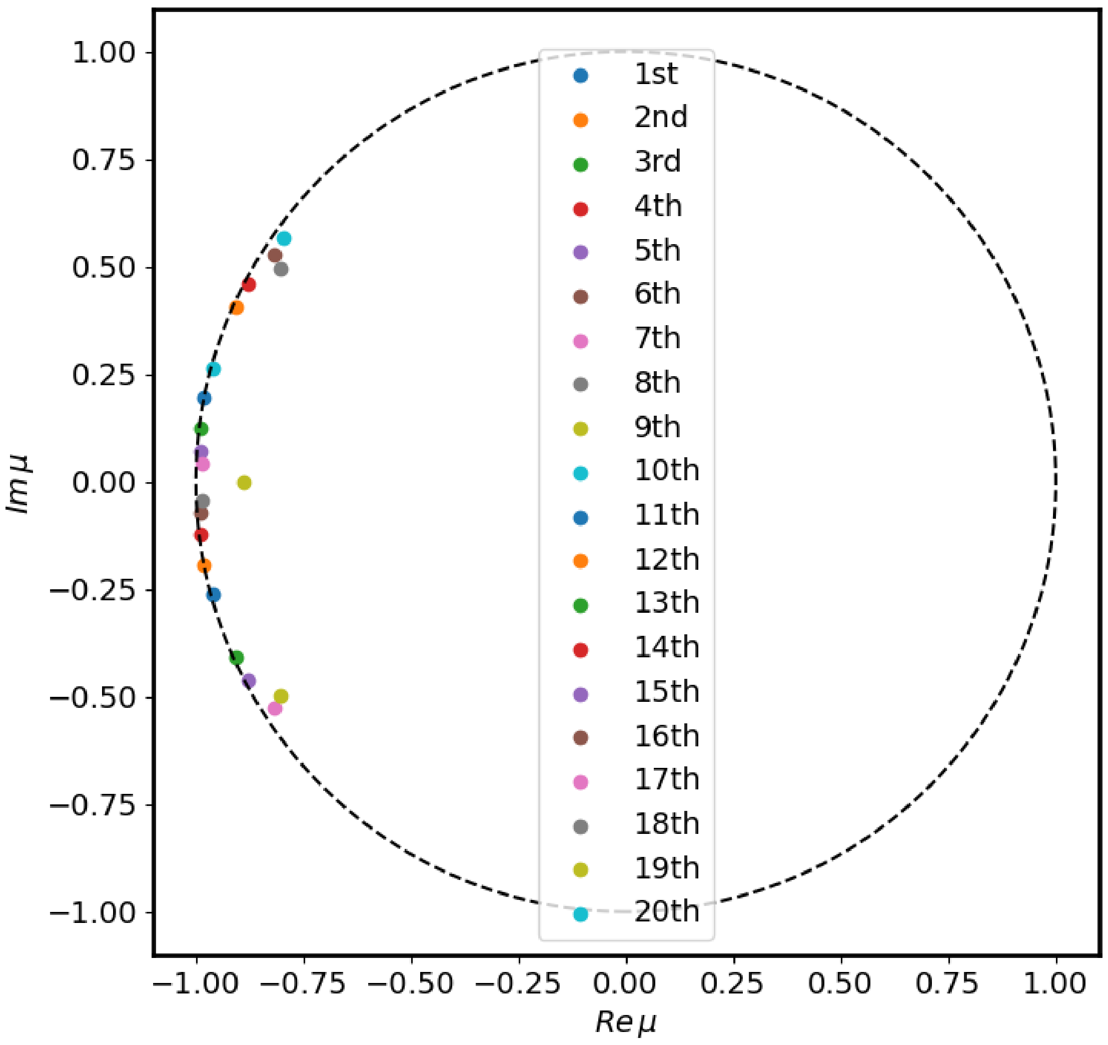

[53]:
```
# compute DMD reconstruction
D2 = dot(Phi, Psi)
#np.allclose(D, D2, atol=1e-10) # True
np.allclose(D, D2) # True
```

[53]: False

[54]:
```
ax = []
plt.clf()
ax_count = 1
max_plot_num = 20

fig = plt.figure(figsize=(18, 60))
fig.subplots_adjust(hspace=1)
```

```
ax.append(fig.add_subplot((max_plot_num), 1, ax_count))
ax[-1].set_xlabel('t')
ax[-1].set_ylabel('y')
ax[-1].set_title('DMD (m0)')
ax[-1].plot(t3, Psi.real[0,:], color='blue', label='Original', marker='x')
ax[-1].plot(t3, Psi.imag[0,:], color='green', label='Complex', marker='x')
ax_count += 1

ax.append(fig.add_subplot((max_plot_num), 1, ax_count))
ax[-1].set_xlabel('t')
ax[-1].set_ylabel('y')
ax[-1].set_title('DMD (m1)')
ax[-1].plot(t3, Psi.real[1,:], color='blue', label='Original', marker='x')
ax[-1].plot(t3, Psi.imag[1,:], color='green', label='Complex', marker='x')
ax_count += 1

ax.append(fig.add_subplot((max_plot_num), 1, ax_count))
ax[-1].set_xlabel('t')
ax[-1].set_ylabel('y')
ax[-1].set_title('DMD (m2)')
ax[-1].plot(t3, Psi.real[2,:], color='blue', label='Original', marker='x')
ax[-1].plot(t3, Psi.imag[2,:], color='green', label='Complex', marker='x')
ax_count += 1

ax.append(fig.add_subplot((max_plot_num), 1, ax_count))
ax[-1].set_xlabel('t')
ax[-1].set_ylabel('y')
ax[-1].set_title('DMD (m3)')
ax[-1].plot(t3, Psi.real[3,:], color='blue', label='Original', marker='x')
ax[-1].plot(t3, Psi.imag[3,:], color='green', label='Complex', marker='x')
ax_count += 1

ax.append(fig.add_subplot((max_plot_num), 1, ax_count))
ax[-1].set_xlabel('t')
ax[-1].set_ylabel('y')
ax[-1].set_title('DMD (m4)')
ax[-1].plot(t3, Psi.real[4,:], color='blue', label='Original', marker='x')
ax[-1].plot(t3, Psi.imag[4,:], color='green', label='Complex', marker='x')
ax_count += 1

ax.append(fig.add_subplot((max_plot_num), 1, ax_count))
ax[-1].set_xlabel('t')
ax[-1].set_ylabel('y')
ax[-1].set_title('DMD (m5)')
ax[-1].plot(t3, Psi.real[5,:], color='blue', label='Original', marker='x')
ax[-1].plot(t3, Psi.imag[5,:], color='green', label='Complex', marker='x')
```

```python
ax_count += 1

ax.append(fig.add_subplot((max_plot_num), 1, ax_count))
ax[-1].set_xlabel('t')
ax[-1].set_ylabel('y')
ax[-1].set_title('DMD (m6)')
ax[-1].plot(t3, Psi.real[6,:], color='blue', label='Original', marker='x')
ax[-1].plot(t3, Psi.imag[6,:], color='green', label='Complex', marker='x')
ax_count += 1

ax.append(fig.add_subplot((max_plot_num), 1, ax_count))
ax[-1].set_xlabel('t')
ax[-1].set_ylabel('y')
ax[-1].set_title('DMD (m7)')
ax[-1].plot(t3, Psi.real[7,:], color='blue', label='Original', marker='x')
ax[-1].plot(t3, Psi.imag[7,:], color='green', label='Complex', marker='x')
ax_count += 1

ax.append(fig.add_subplot((max_plot_num), 1, ax_count))
ax[-1].set_xlabel('t')
ax[-1].set_ylabel('y')
ax[-1].set_title('DMD (m8)')
ax[-1].plot(t3, Psi.real[8,:], color='blue', label='Original', marker='x')
ax[-1].plot(t3, Psi.imag[8,:], color='green', label='Complex', marker='x')
ax_count += 1

ax.append(fig.add_subplot((max_plot_num), 1, ax_count))
ax[-1].set_xlabel('t')
ax[-1].set_ylabel('y')
ax[-1].set_title('DMD (m9)')
ax[-1].plot(t3, Psi.real[9,:], color='blue', label='Original', marker='x')
ax[-1].plot(t3, Psi.imag[9,:], color='green', label='Complex', marker='x')
ax_count += 1

plt.show()
#plt.savefig( 'output_DMD2.png' )
```

```
<Figure size 1000x800 with 0 Axes>
```

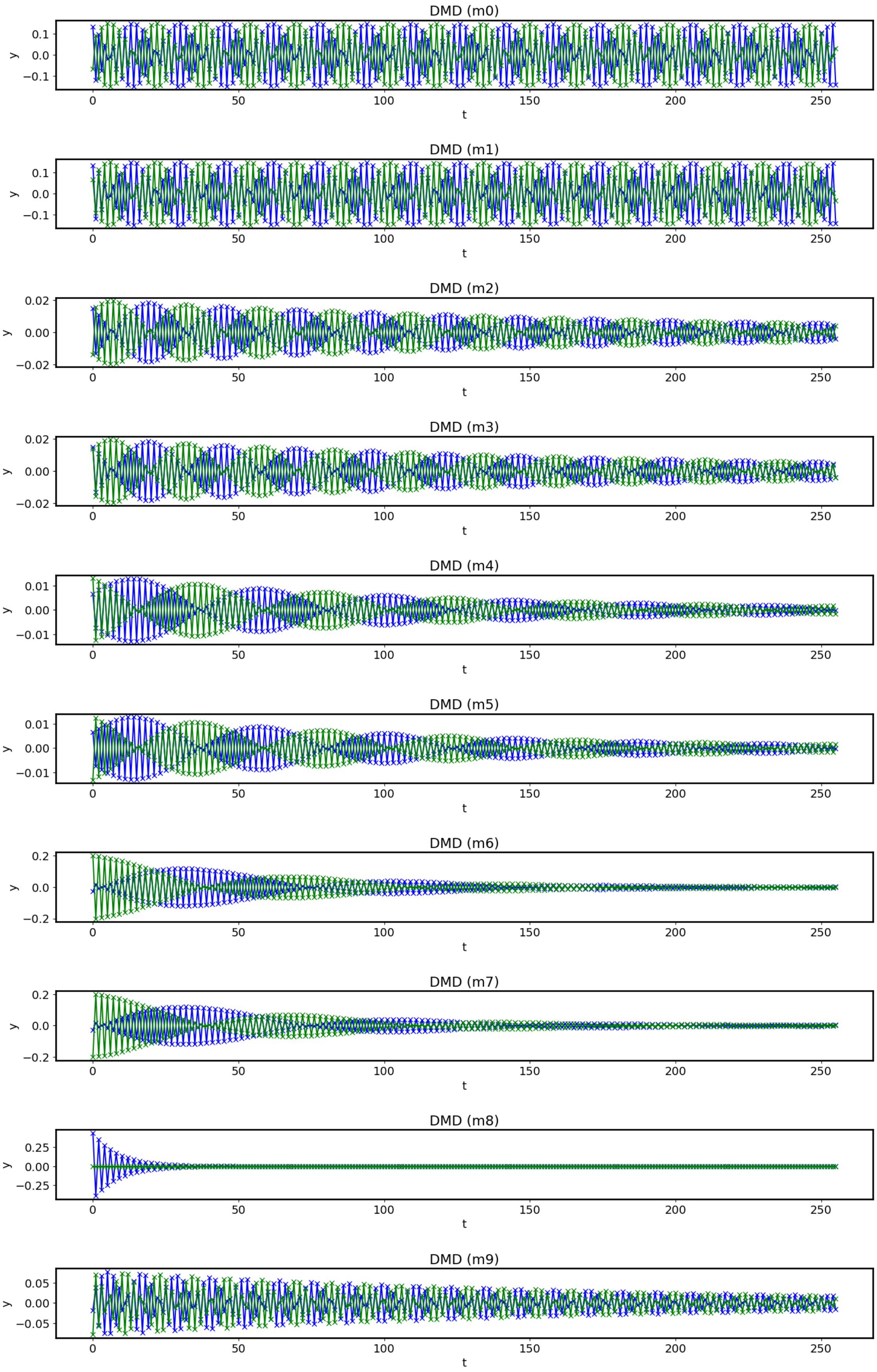
DMD (m0)
DMD (m1)
DMD (m2)
DMD (m3)
DMD (m4)
DMD (m5)
DMD (m6)
DMD (m7)
DMD (m8)
DMD (m9)
y
t
0
50
100
150
200
250

[55]:
```
S = diagsvd(Sig, 128, 255)
```

[56]:
```
print(U@S@Vh)
```

```
[[21.95 29.75 30.05 …  4.45 10.5  19.2 ]
 [29.75 30.05 23.9  … 10.5  19.2  25.15]
 [30.05 23.9  14.05 … 19.2  25.15 25.95]
 …
 [13.5  14.8  14.2  … 46.2  48.65 52.05]
 [14.8  14.2  11.1  … 48.65 52.05 51.45]
 [14.2  11.1   7.15 … 52.05 51.45 42.7 ]]
```

[57]:
```
D_DMD=U@S@Vh
```

[58]:
```
ax = []
plt.clf()

fig = plt.figure(figsize=(18,3))

ax.append(fig.add_subplot(1, 1, 1))
ax[-1].set_xlabel('t')
ax[-1].set_ylabel('y')
ax[-1].set_title('D_DMD')
ax[-1].plot(t1[0:255], D_DMD[0][0:255], color='blue', label='Original',␣
↪marker='.', markersize=3)
ax[-1].set_ylim(-100, 100)

plt.show()
```

```
&lt;Figure size 1000x800 with 0 Axes&gt;
```

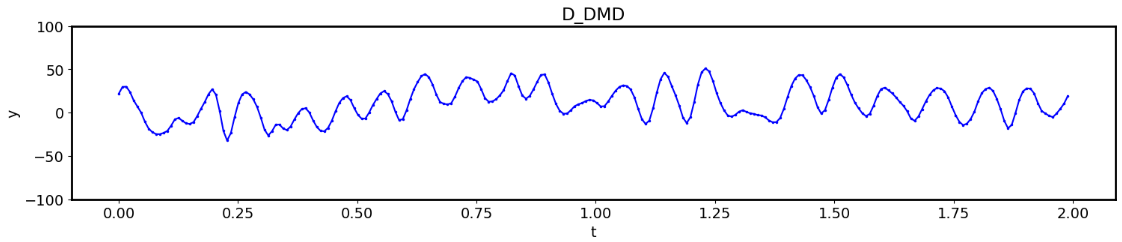

[59]:
```
def X_to_TS(X_i):
    """Averages the anti-diagonals of the given elementary matrix, X_i, and␣
↪returns a time series."""
    # Reverse the column ordering of X_i
```

```
    X_rev = X_i[::-1]
    # Full credit to Mark Tolonen at https://stackoverflow.com/a/6313414 for␣
↪this one:
    return np.array([X_rev.diagonal(i).mean() for i in range(-X_i.shape[0]+1,␣
↪X_i.shape[1])])
```

[60]:
```
# Example elementary matrix
X_i = np.array([[1, 2, 3],
                [4, 5, 6],
                [7, 8, 9]])

# Calling the function
result = X_to_TS(X_i)
print(result)
```

```
[1. 3. 5. 7. 9.]
```

[61]:
```
X_rev = X_i[::-1]
X_rev
```

[61]:
```
array([[7, 8, 9],
       [4, 5, 6],
       [1, 2, 3]])
```

[62]:
```
-X_i.shape[0]+1, X_i.shape[1]
```

[62]:
```
(-2, 3)
```

[63]:
```
X_rev.diagonal(-1)
```

[63]:
```
array([4, 2])
```

[64]:
```
X_rev.diagonal(1)
```

[64]:
```
array([8, 6])
```

[65]:
```
ax = []
plt.clf()
F_all = X_to_TS(X_elem[[0,1,2,3,4,5,6,7,8,9,10,11,12,13,14,15,16,17,18,19]].
↪sum(axis=0))

fig = plt.figure(figsize=(18,3))

ax.append(fig.add_subplot(1, 1, 1))
ax[-1].set_xlabel('t')
ax[-1].set_ylabel('y')
ax[-1].set_title('F_recon')
```

```
#ax[-1].plot(t1[0:128], D2[0], color='green', label='Original', marker='.',␣
 ↪markersize=3)
ax[-1].plot(t1[0:382], F_all[0:382], color='red', label='Original', marker='.',␣
 ↪markersize=3)
#ax[-1].plot(t, F_recon.imag, color='green', label='Complex', marker='.',␣
 ↪markersize=3)
ax[-1].plot(t1[0:382], eeg_384[0:382], color='blue', label='Original', marker='.
 ↪', markersize=3)
ax[-1].set_ylim(-100, 100)

plt.show()
fig.savefig('Sev_case01_F_recon.svg')
```

<Figure size 1000x800 with 0 Axes>

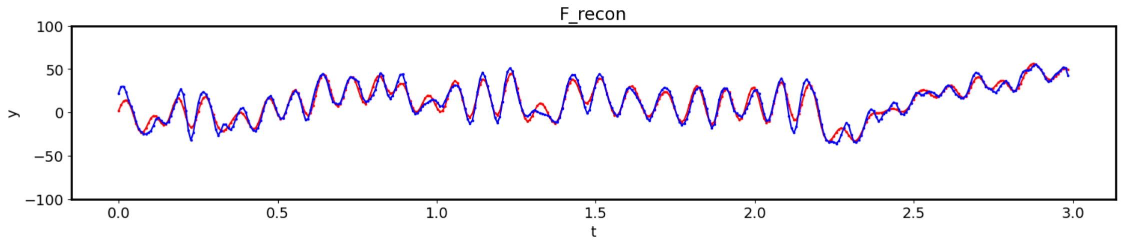

[66]:
```
### CCC
F = eeg_384[:384]

n = min(10,d) # In case of noiseless time series with d < 12.

# Fiddle with colour cycle - need more colours!
ax = []
plt.clf()

fig = plt.figure(figsize=(18,3))

fig = plt.subplot()
color_cycle = cycler(color=plt.get_cmap('tab20').colors)
fig.axes.set_prop_cycle(color_cycle)

# Convert elementary matrices straight to a time series - no need to construct␣
 ↪any Hankel matrices.
for i in range(n):
    F_i = X_to_TS(X_elem[i])
    fig.axes.plot(t1[:382], F_i, lw=2)

fig.axes.plot(t1, F, alpha=1, lw=1)
```

```
fig.set_xlabel("$t$")
fig.set_ylabel(r"$\tilde{F}_i(t)$")
legend = [r"$\tilde{F}_{%s}$" %i for i in range(n)] + ["$F$"]
fig.set_title("The First 9 Components of the EEG Time Series")
fig.legend(legend, loc=(1.05,0.1));
```

<Figure size 1000x800 with 0 Axes>

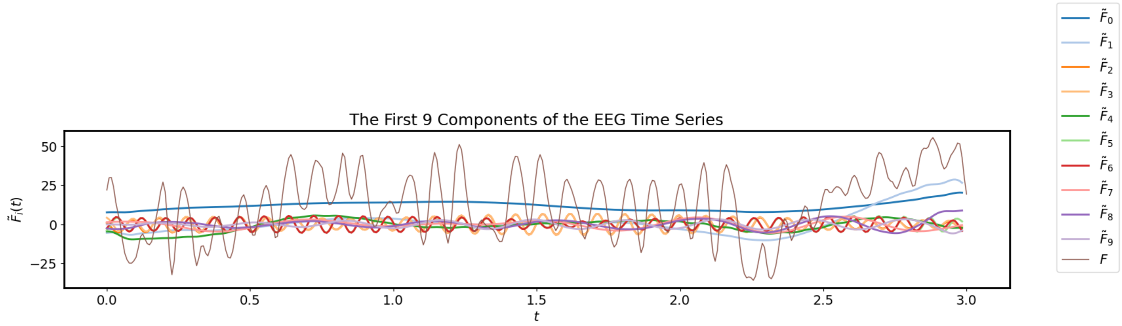

[67]:
```
ax = plt.matshow(D)
plt.xlabel("$L$-Lagged Vectors")
plt.ylabel("$K$-Lagged Vectors")
plt.colorbar(ax.colorbar, fraction=0.025)
ax.colorbar.set_label("$F(t)$")
plt.title("The Trajectory Matrix for the EEG Time Series");
```

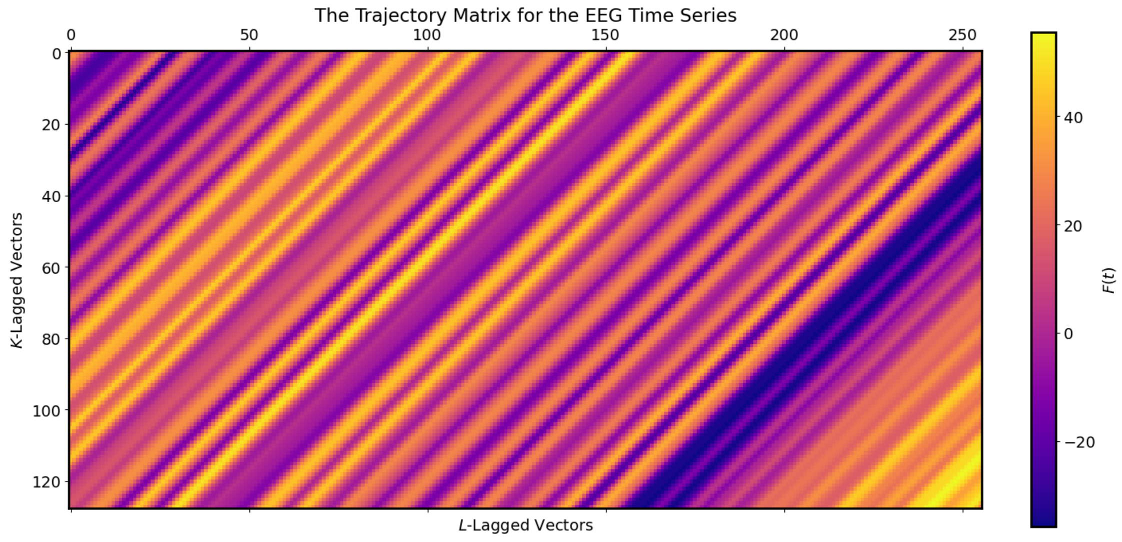

[68]:
```python
ax = plt.matshow(D2.real[:,:])
plt.xlabel("$L$-Lagged Vectors")
plt.ylabel("$K$-Lagged Vectors")
plt.colorbar(ax.colorbar, fraction=0.025)
ax.colorbar.set_label("$F(t)$")
plt.title("The Trajectory Matrix for the EEG Time Series");
plt.savefig('Sev_case01_Trajectory_2.svg')
```

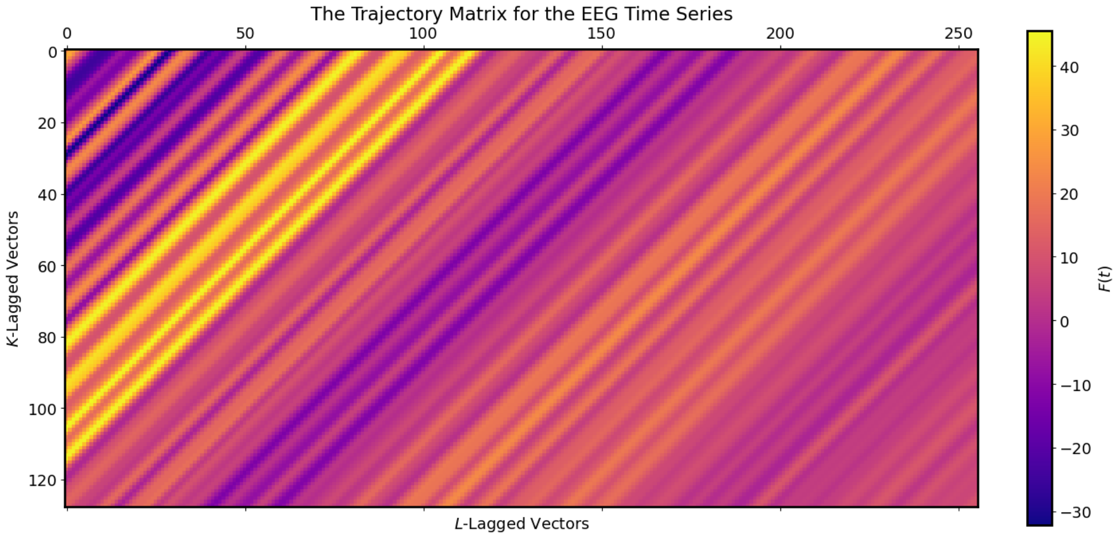

[69]:
```python
#CCC
# Assemble the grouped components of the time series.
F0 = X_to_TS(X_elem[[0,1]].sum(axis=0))
F1 = X_to_TS(X_elem[[2, 3]].sum(axis=0))
F2 = X_to_TS(X_elem[[4,5,6]].sum(axis=0))
F3 = X_to_TS(X_elem[[7,8,9]].sum(axis=0))
F4 = X_to_TS(X_elem[[10,11,12,13,14,15,16,17,18,19]].sum(axis=0))
F5 =␣
  ↪X_to_TS(X_elem[[20,21,22,23,24,25,26,27,28,29,30,31,32,33,34,35,36,37,38,39,40,41,42,43,44,
                                           ␣
  ↪50,51,52,53,54,55,56,67,68,59,60,61,62,63,64,65,66,67,68,69,70,71,72,73,74,75,76,77,78,79,␣
  ↪80,81,82,83,84,
                                          ␣
  ↪85,86,87,88,89,90,91,92,93,94,95,96,97,98,99,100,101,102,103,104,105,106,107,108,109,110,11
                             113,114,115,116,117,118,119,120,121,122,123]].
  ↪sum(axis=0))

# Plot the toy time series and its separated components on a single plot.
ax = []
plt.clf()
```

```
fig = plt.figure(figsize=(18,3))

plt.plot(t1[:256], F[:256], lw=1)
plt.plot(t1[:256], F0[:256])
plt.plot(t1[:256], F1[:256])
plt.plot(t1[:256], F2[:256])
plt.plot(t1[:256], F3[:256])
plt.plot(t1[:256], F4[:256], alpha=0.5)
plt.plot(t1[:256], F5[:256], alpha=0.5)
plt.xlabel("$t$")
plt.ylabel(r"$\tilde{F}^{(j)}$")
#groups = ["trend", "periodic 1", "periodic 2", "noise"]
groups = ["F0", "F1", "F2", "F3", "F4"]
legend = ["$F$"] + [r"$\tilde{F}^{(\mathrm{%s})}$"%group for group in groups]
plt.legend(legend)
plt.title("Grouped Time Series Components")
plt.show()

fig.tight_layout()

fig.savefig('Sev_case01_Grouped.svg')
```

```
<Figure size 1000x800 with 0 Axes>
```

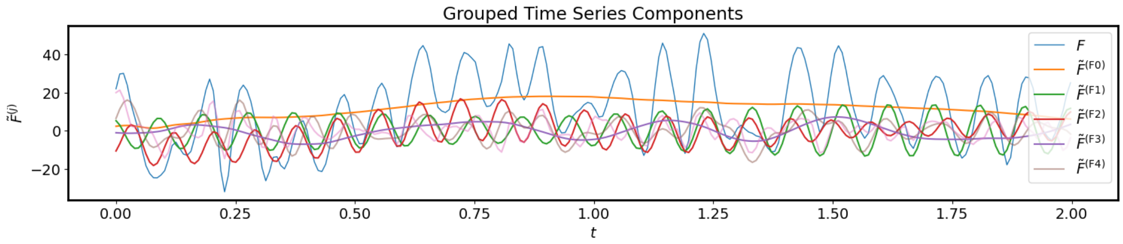

[70]:
```
eeg_recon = []
eeg_recon.append(F0)
eeg_recon.append(F1)
eeg_recon.append(F2)
eeg_recon.append(F3)
eeg_recon.append(F4)
eeg_recon.append(F5)
eeg_recon = np.array(eeg_recon)
eeg_recon_T = eeg_recon.T
```

[71]:
```
import emd
```

[72]:
```
emd.plotting.plot_imfs(eeg_recon_T, cmap=True)
plt.savefig('Sev_case01_dmd.svg')
```

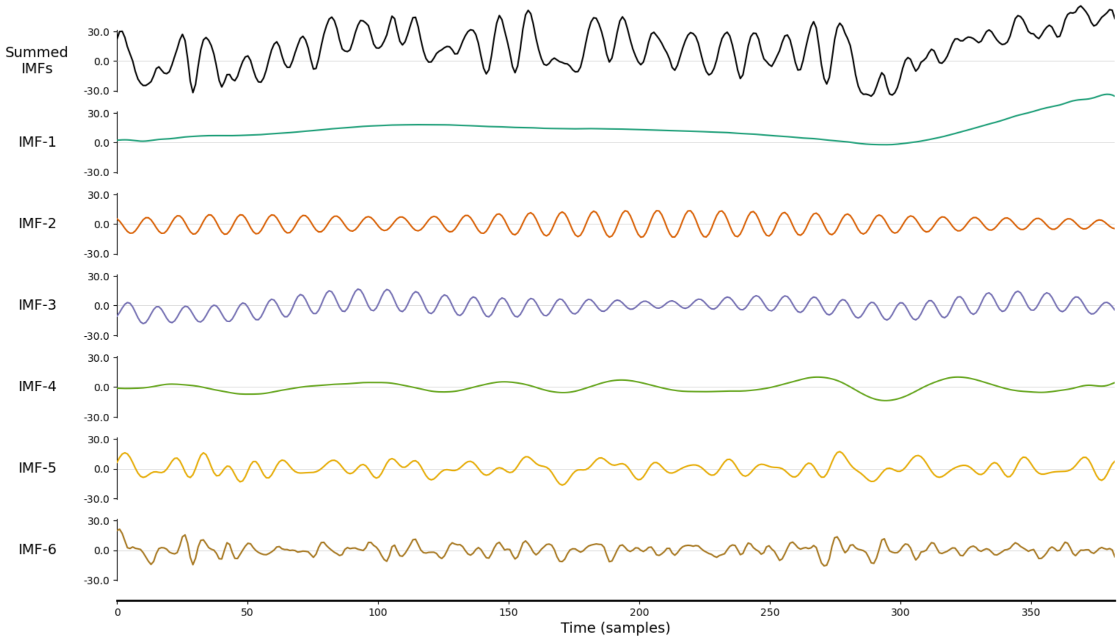

[73]:
```
sample_rate = 128
seconds = 3
num_samples = int(sample_rate * seconds)  #
time_vect = np.linspace(0, seconds, num_samples)
```

[74]:
```
IP, IF, IA = emd.spectra.frequency_transform(eeg_recon_T, sample_rate,␣
 ↪'hilbert')
```

[75]:
```
freq_edges, freq_bins = emd.spectra.define_hist_bins(0.1, 64, 128)
#hht = emd.spectra.hilberthuang(IF, IA, freq_edges)

freq_range = (0, 64, 128)  # 0 to 64Hz in 128 steps
f, hht = emd.spectra.hilberthuang(IF, IA, freq_range, sum_time=False)
```

[76]:
```
freq_edges, freq_centres = emd.spectra.define_hist_bins(0, 100, 128, 'linear')

# Amplitude weighted HHT per IMF
f, spec_weighted = emd.spectra.hilberthuang(IF, IA, freq_edges, sum_imfs=False)

# Unweighted HHT per IMF - we replace the instantaneous amplitude values with␣
 ↪ones
```

```
f, spec_unweighted = emd.spectra.hilberthuang(IF, np.ones_like(IA), freq_edges,␣
 ↪sum_imfs=False)
```

[77]:
```
plt.figure(figsize=(10, 4))
plt.subplots_adjust(wspace=0.4)
plt.subplot(121)
plt.plot(freq_centres, spec_unweighted)
plt.xticks(np.arange(10)*10)
plt.xlim(0, 32)
plt.ylim(0, 400)
plt.xlabel('Frequency (Hz)')
plt.ylabel('Count')
plt.title('unweighted\nHilbert-Huang Transform')

plt.subplot(122)
plt.plot(freq_centres, spec_weighted)
plt.xticks(np.arange(10)*10)
plt.xlim(0, 32)
plt.xlabel('Frequency (Hz)')
plt.ylabel('Power')
plt.title('IA-weighted\nHilbert-Huang Transform')
plt.legend(['IMF-1', 'IMF-2', 'IMF-3', 'IMF-4', 'IMF-5', 'IMF-6'],␣
 ↪frameon=False)
plt.savefig('Sev_case01_HHT_freq.svg')
```

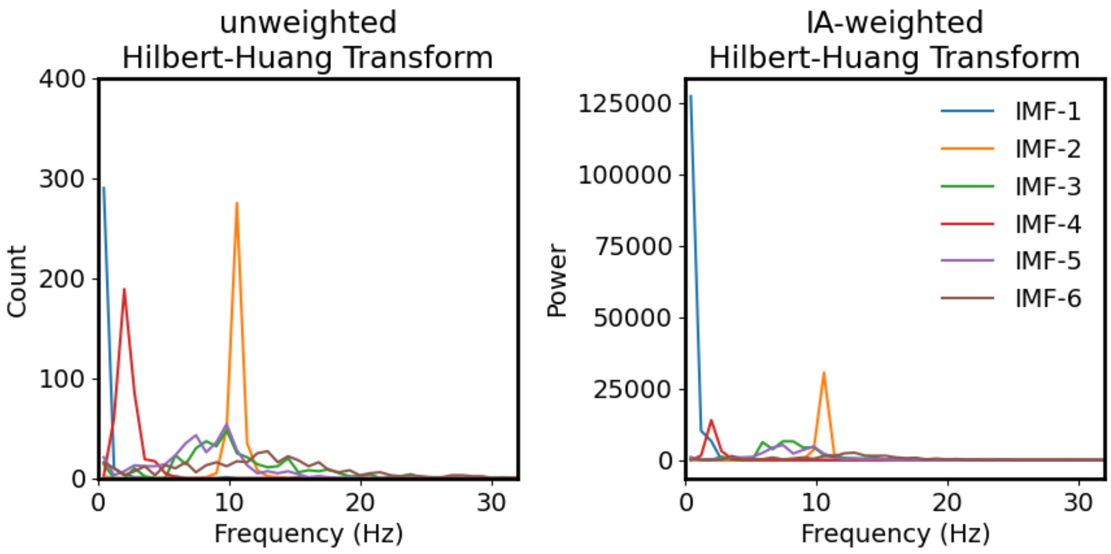

[78]:
```
len(hht[0]), len(time_vect), len(freq_bins)
```

[78]: (382, 384, 128)

[79]:
```
fig = plt.figure(figsize=(6, 4))
emd.plotting.plot_hilberthuang(hht, time_vect, freq_bins,
                                    time_lims=(0, 2.99), freq_lims=(0.1, 32),␣
  ↪vmax=10,
                                    fig=fig, log_y=False)
#plt.savefig('data_eeg_sev1/HHT_spectrum-1.svg')
```

[79]:
```
<Axes: title={'center': 'Hilbert-Huang Transform'}, xlabel='Time',
ylabel='Frequency'>
```

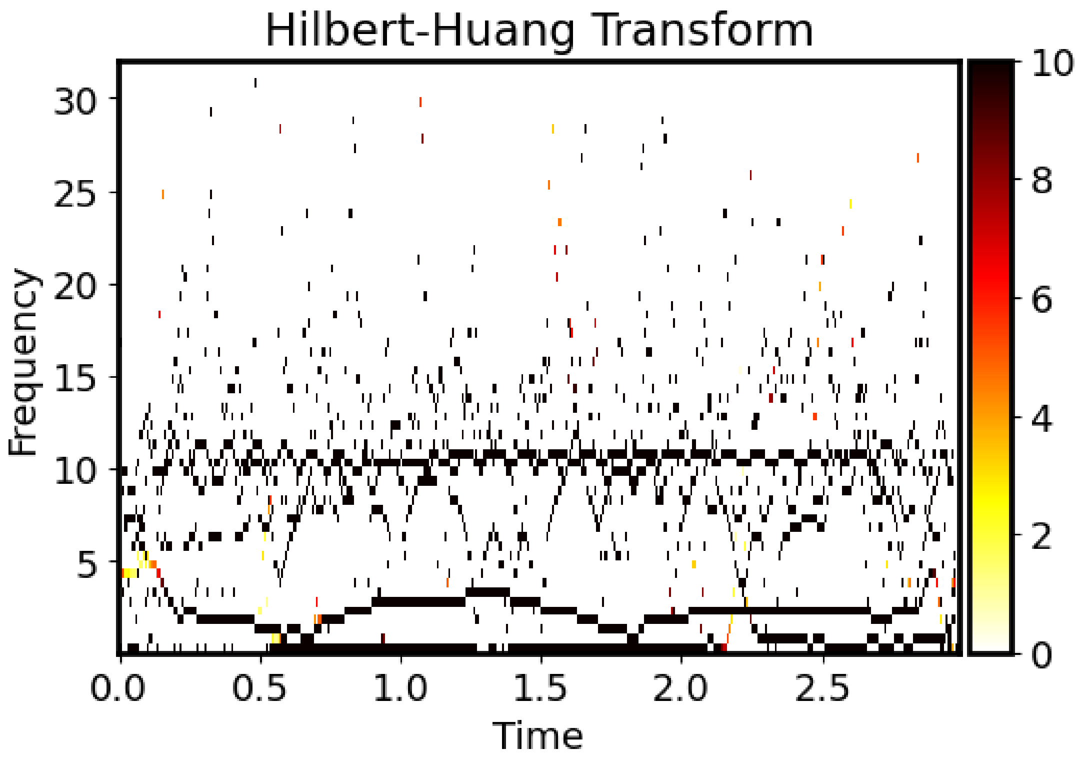

[80]:
```
fig = plt.figure(figsize=(6, 4))
emd.plotting.plot_hilberthuang(hht, time_vect, freq_bins,cmap='ocean_r',
                                    time_lims=(0, 2.99), freq_lims=(0.1, 32),␣
  ↪vmax=10,
                                    fig=fig, log_y=False)
plt.savefig('Sev_case01_HHT_spectrum.svg')
```

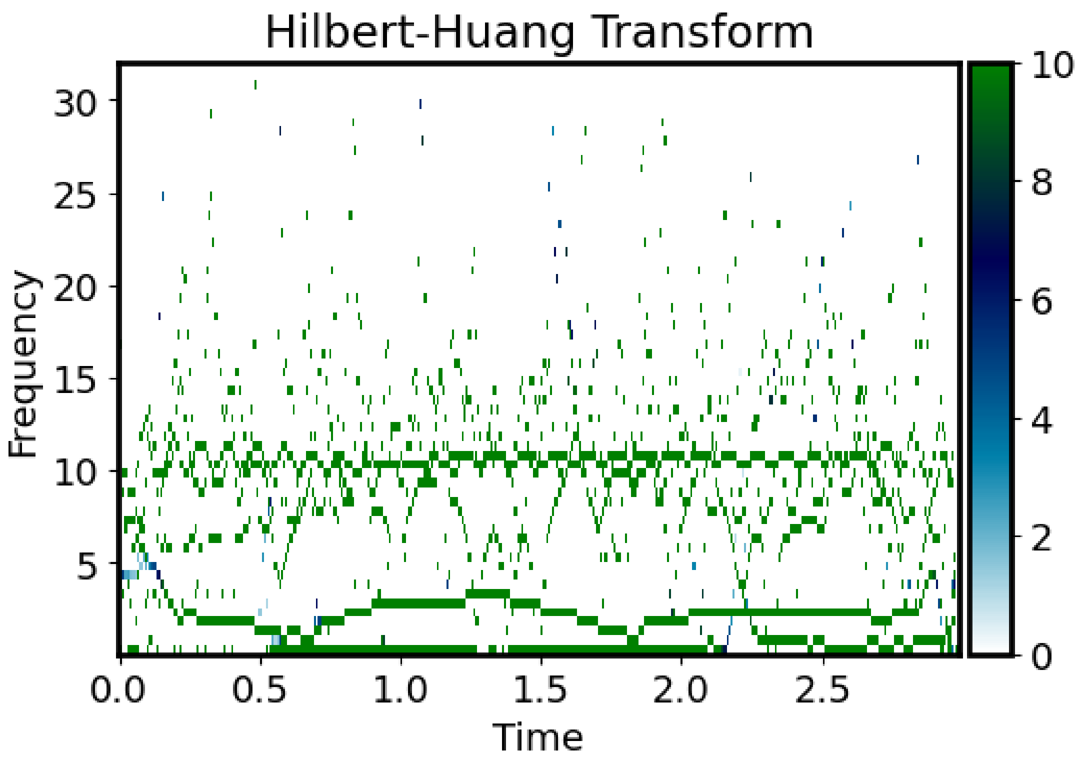

[81]:
```
ax = []
plt.clf()

fig = plt.figure(figsize=(18,10))
fig.subplots_adjust(hspace=1)

ax.append(fig.add_subplot(2, 1, 1))
ax[-1].set_xlabel('t')
ax[-1].set_ylabel('y')
#ax[-1].set_title('F')
ax[-1].plot(t1[:382], F[:382], lw=1, color='blue', label='Original', marker='.
↪', markersize=3)
ax[-1].plot(t1[:382], F_all[:382], color='red', label='Original', marker='.',␣
↪markersize=3)
ax[-1].set_ylim(-100, 100)
legend = ["$F$"]+ ["$F recon$"]
plt.legend(legend)
plt.title("Original Time Series Components")

ax.append(fig.add_subplot(2, 1, 2))
ax[-1].set_xlabel('t')
ax[-1].set_ylabel('y')
```

```
ax[-1].set_title('DMD')
ax[-1].plot(t1[:382], F0[:382], lw=1, color='orange', label='Original', marker='.', markersize=3)
ax[-1].plot(t1[:382], F1[:382], lw=1, color='green', label='Original', marker='.', markersize=3)
ax[-1].plot(t1[:382], F2[:382], lw=1, color='purple', label='Original', marker='.', markersize=3)
ax[-1].plot(t1[:382], F3[:382], lw=1, color='red', label='Original', marker='.', markersize=3)
ax[-1].plot(t1[:382], F4[:382], lw=1, color='brown', label='Original', marker='.', markersize=3)
ax[-1].set_ylim(-100, 100)
plt.xlabel("$t$")
plt.ylabel(r"$\tilde{F}^{(j)}$")
#groups = ["trend", "periodic 1", "periodic 2", "noise"]
groups = ["F0", "F1", "F2", "F3","F4"]
#legend = ["$F$"] + [r"$\tilde{F}^{(\mathrm{%s})}$"%group for group in groups]
legend = [r"$\tilde{F}^{(\mathrm{%s})}$"%group for group in groups]
plt.legend(legend)
plt.title("Grouped Time Series Components")

plt.show()
```

```
<Figure size 1000x800 with 0 Axes>
```

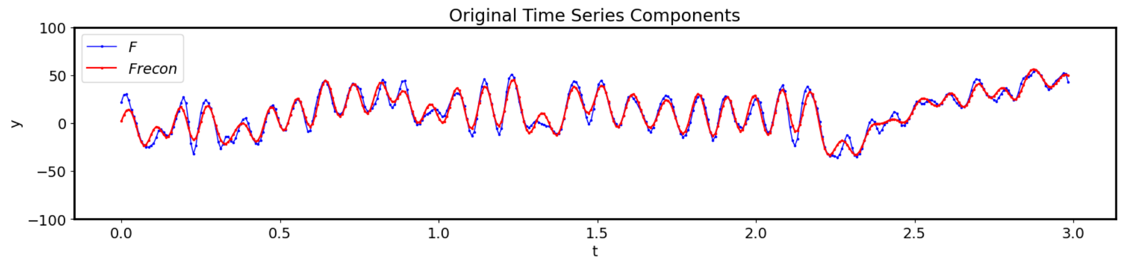

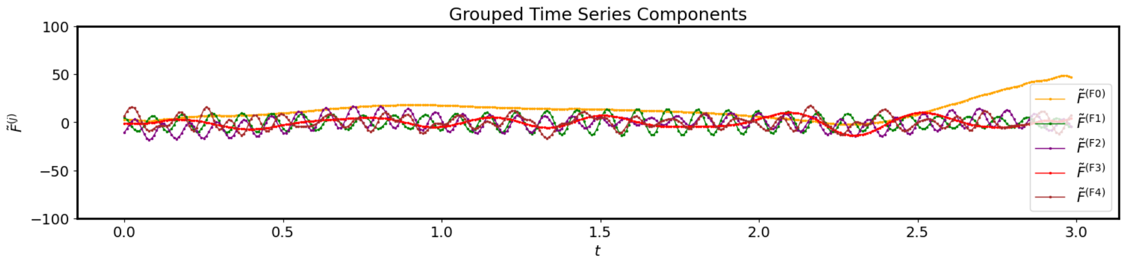

Supplement: Supplementary file 1 [file sensors-26-01212-s001.zip › File01_python_code.pdf]
